# Supplementary material for: Bridging the gap: A new species of arboreal Abronia (Squamata: Anguidae) from the Northern Highlands of Chiapas, Mexico
Source: PLoS One. 2024 Jan 3;19(1):e0295230. doi: 10.1371/journal.pone.0295230 (PMC10763973; doi:10.1371/journal.pone.0295230)
Supplement: S1 Appendix — (PDF) [file pone.0295230.s001.pdf]

Appendix S1. Complete Spanish translation of article.
Apéndice S1. Traducción completa al español del artículo.

Cerrando la brecha: Una nueva especie de *Abronia* arborícola (Squamata: Anguidae) de las Montañas del Norte de Chiapas, México

Adam G. Clause[1*], Roberto Luna-Reyes,[2¶] Oscar M. Mendoza-Velázquez[3¶], Adrián Nieto-Montes de Oca[4¶], Israel Solano-Zavaleta[5¶]

[1] Department of Herpetology, San Diego Natural History Museum, San Diego, California, Estados Unidos

[2] Dirección de Áreas Naturales y Vida Silvestre, Secretaría de Medio Ambiente e Historia Natural, Tuxtla Gutiérrez, Chiapas, México

[3] Instituto de Ciencias Biológicas, Universidad de Ciencias y Artes de Chiapas, Tuxtla Gutiérrez, Chiapas, México

[4] Departamento de Biología Evolutiva, Facultad de Ciencias, Universidad Nacional Autónoma de México, Ciudad de México, México

[5] Departamento de Ecología y Recursos Naturales, Facultad de Ciencias, Universidad Nacional Autónoma de México, Ciudad de México, México

* Autor de correspondencia
Correo: adamclause@gmail.com (AGC)

[¶] Estos cuatro autores contribuyeron de igual forma en este trabajo; se enlistan alfabéticamente por apellido.

Resumen

Los bosques de montaña de América Central son reconocidos por su biodiversidad endémica, y los dragoncitos (género *Abronia*) son vertebrados de alto perfil que son endémicos de esta región. En este trabajo, describimos una nueva especie de *Abronia* arborícola que solo se ha registrado en la localidad tipo en las Montañas del Norte de Chiapas, México. La nueva especie se puede distinguir de todos los demás miembros del género *Abronia* por la siguiente combinación de caracteres: la ausencia de escamas supra-auriculares protuberantes o semejantes a espinas, la ausencia de escamas posterolaterales de la cabeza protuberantes o similares al borde de un casco, dorso de la cabeza de color amarillo pálido con distintivas marcas oscuras, de 35–39 hileras transversales de escamas dorsales, escamas de la primera hilera de ventrales más grandes en relación con el tamaño de las escamas de la hilera medial adyacente, y el dorso marrón con bandas transversales más oscuras que a veces se reducen a hileras de manchas. Provisionalmente hemos incluido a la nueva especie en el subgénero *Lissabronia* basándonos en evidencia genómica y morfológica, pero nuestros resultados también sugieren una estrecha relación con el subgénero *Abaculabronia*. La nueva especie está separada geográficamente de las especies de *Lissabronia* y *Abaculabronia* más cercanas por las tierras bajas de la Depresión Central de Chiapas. La pérdida continua de hábitat y otros factores ponen en riesgo a la nueva especie, lo que nos lleva a proponer su inclusión en múltiples listados de especies amenazadas. Debido a que las Montañas del Norte tienen poca cobertura en áreas naturales protegidas, comentamos brevemente sobre el potencial de este nuevo dragoncito para estimular la conservación en la región.

Introducción

Los bosques de las tierras altas de América Central son áreas de biodiversidad más o menos análogas a las islas [1, 2]. Estos bosques frescos y húmedos forman un archipiélago de parches separados rodeados por mares de vegetación de tierras bajas [3, 4]. Este aislamiento ha fomentado la evolución de una notable diversidad de organismos [5]. Las causas hipotéticas de esta diversidad siguen estando pobremente comprobadas, pero probablemente intervienen factores biológicos, geológicos y climáticos interrelacionados [5, 6]. Muchos autores concuerdan en que complejos procesos históricos de formación de montañas, junto a eventos paleoclimáticos, han creado barreras biogeográficas correlacionadas con la especiación de muchos vertebrados de América Central [7-13]. Actualmente, especies emblemáticas y llamativas de aves [14, 15], serpientes [16, 17], ranas [18, 19] y salamandras [20, 21] únicamente viven en estos bosques de montaña. Muchos grupos de reptiles y anfibios adaptados a estos bosques tienen tasas notablemente rápidas de recambio de especies en distancias cortas, con especies que a menudo se conocen solo a partir de un único pico o macizo aislado [18, 22-27]. Estas altas tasas de endemismo, junto con amenazas apremiantes como el cambio climático y la

rápida deforestación causada por los humanos [28-31], han generado mucho interés en la conservación de las tierras altas de América Central [32-35].

Un grupo relevante para la conservación que se ha diversificado en estos bosques de "islas virtuales" son los dragoncitos del género *Abronia* Gray, 1838 [36] (Anguidae: Gerrhonotinae). Aunque existe un registro de California, Estados Unidos de una especie extinta del Mioceno referida a este género [37], todos los dragoncitos (género *Abronia*) actualmente se distribuyen acumulativamente desde el centro y este de México hacia el sur hasta el oeste de Panamá [38]. Estas lagartijas casi siempre están restringidas a los bosques húmedos de las tierras altas, siendo los bosques mesófilos de montaña y los bosques estacionales secos de pino-encino los hábitats particularmente comunes [39]. Tradicionalmente el género incluía solo especies arborícolas, aunque la modalidad arborícola es una presunción para algunas especies porque no existen datos ecológicos para ellas [39-42]. Sin embargo, recientes análisis genómicos indican que este conjunto de especies arborícolas y probablemente arborícolas es un grupo parafilético con respecto al género estrictamente terrestre *Mesaspis* Cope, 1877 [38, 43]. Debido a que *Abronia* tiene prioridad nomenclatural sobre *Mesaspis*, los autores de ese estudio recomendaron la sinonimización de este último [38]. En este trabajo seguimos ese arreglo taxonómico. Historias taxonómicas más exhaustivas de *Abronia* y el anterior género *Mesaspis* están disponibles en otros trabajos [38, 44, 45]. Actualmente existen 41 especies reconocidas de *Abronia* [45]. Estas se dividen en 11 clados o grupos de especies. Ocho de estos están compuestos exclusivamente por especies arborícolas, mientras que los tres restantes incluyen solo especies terrestres [38, 39]. Las especies arborícolas (que constituyen más de las tres cuartas partes del género) a menudo son coloridas, icónicas y están en riesgo [46]. La mayoría de las especies arborícolas de *Abronia* también son misteriosas, con distribuciones geográficas limitadas y un comportamiento críptico que se combinan para explicar los pocos avistamientos [39]. Estos factores han convertido a los dragoncitos arborícolas en un objetivo de gran interés para la gestión internacional de la biodiversidad [47-49]. Por lo tanto, es probable que el descubrimiento de especies previamente desconocidas del género *Abronia* motive un fuerte interés por la conservación.

En el estado más al sur de México, las montañas de Chiapas siguen siendo fuentes especialmente prometedoras de diversidad herpetofaunística no documentada. Recientemente en estas áreas de Chiapas se produjeron extensiones de distribución y los primeros registros para México de una culebra y una salamandra poco frecuente de observar [50, 51], además de una especie de *Abronia* arborícola endémica y completamente nueva para la ciencia [44]. Una región probable para hallazgos adicionales de este tipo es el sistema poco explorado de macizos montañosos en la parte norte-central del estado. Estas complejas montañas generalmente se identifican como parte de la región fisiográfica de las Montañas del Norte (Northern Highlands) [52-56]. Sin embargo, también han sido llamadas las Montañas del Centro (Central Highlands) [57], o han sido consideradas parte de la Meseta Central [18], o parte de Los Altos de Chiapas [32]. Independientemente de cómo se nombren estas tierras altas, únicamente se han reportado dos especies de dragoncitos arborícolas allí. La primera es una población no asignada del clado

Scopaeabronia de la Zona Sujeta a Conservación Ecológica "Laguna Bélgica", en la parte suroeste de la región [38, 39, 58, 59]. La segunda es *A. lythrochila*, que pertenece al clado *Auriculabronia* y apenas ingresa a las Montañas del Norte a lo largo de un acantilado al sur cerca del pueblo de Jitotol [60-62]. La mayor parte de las Montañas del Norte son, por lo tanto, una brecha en el mapa de distribución de los dragoncitos arborícolas del género *Abronia*.

En el año 2014, surgieron fotografías intrigantes de una *Abronia* arborícola en esta brecha geográfica. Posteriormente, realizamos expediciones de recolección en los años 2015, 2021, y 2022, y finalmente reunimos suficiente material comparativo. Con base en un análisis integrador de evidencias genómicas, morfológicas y biogeográficas, en este estudio delimitamos esta población como una nueva especie. Sorprendentemente, esta nueva especie no pertenece ni al clado de *Scopaeabronia* ni de *Auriculabronia*. En cambio, encontramos que está estrechamente relacionado con *A. morenica*, que se encuentra a unos 110 km de distancia en dirección opuesta y a través de las inhóspitas tierras bajas de la Depresión Central de Chiapas [44]. También comentamos las implicaciones biogeográficas y de conservación de la nueva especie.

Materiales y métodos

Recolección de especímenes de campo

Durante nuestro trabajo de campo de varios años en las Montañas del Norte de Chiapas, México, capturamos cinco especímenes de la presunta nueva especie de *Abronia* arborícola. Todos los ejemplares provienen de las cercanías del pueblo de Coapilla. Realizamos la búsqueda de ejemplares a pie y trepando a los árboles, y los capturamos con la mano o con un lazo atado a una vara telescópica. Dentro de los cinco días posteriores a la recolección en campo, sacrificamos los ejemplares con una inyección intracardiaca de pentobarbital sódico, recolectamos una muestra de hígado para análisis moleculares posteriores (ver más abajo), luego los fijamos en una dilución al 10% de formalina amortiguada y luego los transferimos a una disolución de etanol al 70% para su almacenamiento permanente. Depositamos los ejemplares en la colección herpetológica del Museo de Zoología "Alfonso L. Herrera" de la Facultad de Ciencias, Universidad Nacional Autónoma de México (MZFC-HE, antes MZFC), o del Museo de Zoología de la Facultad de Estudios Superiores Zaragoza, Universidad Nacional Autónoma de México (MZFZ). Además, examinamos especímenes relevantes alojados en el Museo de Zoología de la Universidad de Ciencias y Artes del Estado de Chiapas (MZ-UNICACH) para confirmar el ensamble de reptiles y anfibios que coexisten con la nueva especie. Los Comités Institucionales de Cuidado y Uso de Animales (*Institutional Animal Care and Use Committees*) no existen en México ni en la institución del autor principal, pero todos nuestros procedimientos de manejo de animales vivos siguieron las recomendaciones disponibles en las Directrices para el Uso de Anfibios y Reptiles Vivos en Investigación de Campo y Laboratorio (*Guidelines for Use of Live Amphibians and Reptiles in Field and Laboratory Research*) [63]. La recolección de especímenes fue autorizada bajo el permiso FAUT-0093 (SGPA/DGVS/4755/19) emitido por la Secretaría de Medio Ambiente y Recursos Naturales a Adrián Nieto-Montes de Oca, y el permiso FAUT-0243 (SGPA/DGVS/03937/21) emitido a Uri Omar García-Vázquez. Los sitios

que visitamos durante el trabajo de campo son propiedad comunal del Ejido de Coapilla, y agradecemos al Comisariado Ejidal (David Cruz-Pérez) y a otros residentes de Coapilla por autorizarnos el acceso a sus tierras para este estudio.

Procedimientos moleculares

Bibliotecas de ddRADseq

Para investigar la distinción y las relaciones filogenéticas de la presunta nueva especie usando evidencia molecular, recopilamos datos de ddRADseq publicados previamente para taxones representativos de los principales clados de *Abronia* [38, 45] y ampliamos este conjunto de datos con nuevos datos de ddRADseq de muestras de tres taxones previamente ausente de las hipótesis filogenéticas moleculares del género: dos especímenes de la presunta nueva especie de Coapilla, el holotipo y dos paratipos de *A. morenica*, y un paratipo de *A. ornelasi*. También generamos nuevos datos ddRADseq para dos especímenes de *Gerrhonotus* y uno de *Elgaria*, así como uno de *A. antauges* (Apéndice S2). Nuestro muestreo incluyó todos los taxones de *Abronia* que ocurren dentro de los 200 km de la presunta nueva especie.

Generamos los nuevos datos ddRADseq siguiendo un procedimiento publicado recientemente utilizado para *Abronia* [45], excepto que extrajimos el ADN genómico de las muestras de la presunta nueva especie, *Gerrhonotus* y *Elgaria* con un DNeasy Blood & Tissue Kit (Quiagen, cat. No. 69504). Además, secuenciamos las muestras de la presunta nueva especie usando una plataforma HiSeqX de extremo emparejado de 150 pb (Macrogen Inc., Corea), mientras que secuenciamos las muestras de *Gerrhonotus* y *Elgaria* usando una plataforma HiSeq de extremo emparejado de 100 pb en la Arizona State University. Todos los nuevos datos de secuencias están disponibles en GenBank (BioProject número de acceso PRJNA 1034133; Apéndice S2).

Procesamos el conjunto de datos ddRADseq usando ipyrad ver. 0.9.50 [64]. Utilizamos configuraciones de parámetros predeterminadas para completar ensamblajes, excepto por las siguientes configuraciones: número máximo de discrepancias de códigos de barras = 1; filtro para adaptadores/códigos de barras = 2 (estricto); tanto la profundidad mínima a la que se realizan las llamadas de base de reglas estadísticas y de mayoría durante la llamada de base de consenso = 10; y umbral de agrupamiento = 0.94 [38, 45]. Además de utilizar la configuración predeterminada de un número mínimo de muestras que deben tener datos en un locus determinado para que se conserven en el ensamblaje final (= cobertura de taxón) de 4, generamos un ensamblaje con cobertura de taxón = 17 (aproximadamente la mitad del número total de muestras, n = 35) para explorar el efecto sobre la solidez de los análisis filogenéticos de diferentes números de loci y diferentes porcentajes de datos faltantes.

Análisis filogenéticos, genética de poblaciones y de delimitación de especies

Realizamos análisis de máxima verosimilitud (ML, por sus siglas en inglés) utilizando RAxML ver. 8.2.12 [65] de las dos matrices con diferente cobertura taxonómica (ver arriba). Las matrices incluyeron todos los loci concatenados con SNP y sitios invariantes para mejorar la

longitud de las ramas y la precisión topológica en las reconstrucciones filogenéticas [66]. Realizamos una búsqueda simultánea para obtener el árbol de ML con mejor puntaje y un análisis de arranque rápido con el modelo GTR + GAMMA, utilizando 100 réplicas de arranque a partir de árboles de secuencia de adición aleatoria. Realizamos todos los análisis en el clúster informático de alto rendimiento Mana de la University of Hawái. Usamos FigTree ver. 1.4.4 [67] para producir la figura con el árbol filogenético resultante.

Caracterizamos la estructura genética de la población dentro de los linajes en el clado compuesto por la presunta nueva especie, *A. morenica* y *A. ornelasi* (ver más abajo) usando conStruct ver. 1.0.5 [68]. El software de conStruct es un método estadístico relativamente nuevo para la inferencia simultánea de patrones continuos y discretos de la estructura de la población. Elegimos este método porque el aislamiento por distancia, un patrón que se distribuye continuamente a lo largo de un paisaje, está muy extendido por naturaleza y porque los modelos de estructura de población discreta pueden atribuir incorrectamente (especialmente cuando el muestreo es discontinuo) la diferenciación debido a procesos continuos como el aislamiento por distancia, a procesos discretos como barreras geográficas, ecológicas o reproductivas entre poblaciones [69]. Debido a que conStruct es sensible a los datos faltantes, primero generamos un conjunto de SNP (uno por locus) en ipyrad ver. 0.9.50 [64] con solo aquellos loci con datos de todas las muestras (6) y otras configuraciones de parámetros como se describe anteriormente. Luego realizamos un análisis de validación cruzada con dos repeticiones, número de capas (K) = 1–4, dos cadenas y 30000 iteraciones para determinar el soporte estadístico para modelos con diferentes números de capas con y sin componente espacial, y finalmente realizamos un análisis para el modelo con mayor soporte estadístico con dos cadenas y 30000 iteraciones.

Realizamos un análisis de delimitación de especies en el clado compuesto por la presunta nueva especie, *A. morenica* y *A. ornelasi* (ver más abajo) utilizando el criterio heurístico para la delimitación de especies basado en un índice de divergencia genealógica (gdi, por sus siglas en inglés) entre poblaciones [70]. Primero, generamos un ensamblado en ipyrad ver. 0.9.50 [64] con solo aquellos loci para los cuales todas las muestras en el ensamblaje (6) tenían datos y otras configuraciones como se describe anteriormente. Luego realizamos un análisis A00 en el programa BPP ver. 4.6.2 [71, 72] para estimar los parámetros de tiempos de divergencia de especies y tamaños poblacionales bajo el modelo coalescente multiespecífico, y finalmente utilizaron las medias posteriores de los parámetros generados por BPP para calcular los gdis [70]. Sobre la base de la distancia genética por pares media no corregida (distancia p) dentro y entre los taxones, asignamos a los parámetros de tamaño de la población (Øs) el IG previo gamma inverso (3, 0.0016), con una media de 0.0016/(3 – 1) = 0.0008; sobre la base de la distancia p media entre el taxón más basal (*A. ornelasi*) y los otros taxones, asignamos el tiempo de divergencia en la raíz del árbol de especies (TAU_0) la gamma inversa anterior IG (3, 0.0016), con media 0.008; usamos la distribución uniforme de Dirichlet para especificar los otros parámetros de tiempo de divergencia [73]. Realizamos el análisis A00 con datos de 1000 loci. Ejecutamos el análisis para 1000000 de generaciones con un período de quemado de 40000 generaciones y muestreando cada 200 generaciones. Para verificar la convergencia, usamos

Tracer ver. 1.7.2 [74]. Realizamos el análisis dos veces para confirmar la consistencia entre las ejecuciones.

Morfología

Consultamos obras de referencia estándar para la terminología de las escamas [75] y para protocolos de conteo de escamas [39, 76]. Anotamos los caracteres bilaterales en ambos lados y referimos las condiciones en los lados izquierdo y derecho como izquierda/derecha. Para los conteos de hileras de escamas transversales dorsales y ventrales, expresamos estos conteos como un intervalo para especímenes con fisión/fusión aberrante de hileras de escamas. Después de la eutanasia, pero antes de la fijación con formalina, tomamos medidas lineales con un Vernier (con 1 mm de precisión), y medimos el peso con una báscula de plato digital (con 0.1 g de precisión).

Basamos nuestro diagnóstico y comparaciones con otros miembros del género *Abronia* mediante una revisión de la literatura descriptiva relevante, con detalles disponibles en otros trabajos [45]. Para facilitar la comparación con la literatura anterior, el formato de nuestra descripción se basa en gran medida en la última monografía sobre *Abronia* [39]. De los 11 clados o grupos de especies de *Abronia* reconocidos por el tratamiento morfológico más reciente [39] y/o tratamiento molecular [38], todos están respaldados por caracteres morfológicos y todos excepto tres también están respaldados por datos moleculares. Sin embargo, cuatro de estos grupos carecen de un nombre formal y todos los clados terrestres son morfológicamente similares. Para facilitar la comparación en el diagnóstico, nos referimos a los seis grupos de especies nombrados (subgéneros) de *Abronia* arborícola que se han reconocido tradicionalmente [39], y nos referimos a todas las *Abronia* terrestres como "miembros anteriores del género *Mesaspis*". Aunque este arreglo refleja imperfectamente la historia evolutiva dentro de *Abronia* [38], estos grupos son morfológicamente cohesivos y fácilmente diagnosticables [39]. Por lo tanto, este arreglo facilita la comparación de manera más simple y directa de la presunta nueva especie con todas las especies congenéricas existentes. Además, en este estudio adoptamos el concepto de especie evolutiva [77-79] y seguimos un enfoque integrador que usa distinción genética, diferencias fijas en las características morfológicas [80] y el aislamiento geográfico para delimitar la existencia de distintos linajes a nivel de especie.

Acto Nomenclatural

La edición electrónica de este artículo cumple con los requisitos del Código Internacional de Nomenclatura Zoológica enmendado (ICZN por sus siglas en inglés), y por consiguiente los nuevos nombres contenidos en este documento están disponibles bajo ese Código en la edición electrónica de este artículo. Este trabajo publicado y el acto de nomenclatura que contiene han sido registrados en ZooBank, el sistema de registro en línea para el ICZN. El LSID (Life Science Identifier) de ZooBank se puede resolver y la información asociada se puede ver a través de cualquier navegador web estándar agregando el LSID al prefijo http://zoobank.org/. El LSID para esta publicación es urn:lsid:zoobank.org:pub:5E56D78F-1DEF-42B3-AEBD-

B2AD2E1D1817. La edición electrónica de este trabajo fue publicada en una revista con ISSN, ha sido archivada y está disponible en el repositorio digital LOCKSS.

Resultados

El número total de lecturas sin procesar que obtuvimos de las bibliotecas de ddRADseq generadas para este estudio (ver arriba) fue el siguiente: 4195570 y 3557445 para las dos muestras de la presunta nueva especie de Coapilla (MZFC-HE 36544 y 36545, respectivamente); 2388811, 3053286 y 2317826 para las tres muestras de *A. morenica* (MZFC-HE 33486, 33487 y 33490, respectivamente); 130062 y 3552001 para las muestras de *A. antauges* (MZFC-HE 29310) y *A. ornelasi* (UTA R-12499), respectivamente; y 1876800, 2771566 y 3267734 para las muestras de *Gerrhonotus liocephalus* (MZFC-HE 16988), *G. mccoyi* (MZFC-HE 29648) y *Elgaria kingii* (ANMO 4163), respectivamente.

Los ensamblajes compuestos por loci con datos de al menos 4 y 17 muestras tenían secuencias de 27527 y 3425 loci, 83388 y 22000 sitios informativos de parsimonia, tamaños de matriz de secuencia de 3846667 y 488700 sitios, y 74.18% y 39.18% de sitios faltantes, respectivamente.

Las hipótesis filogenéticas recuperadas de los análisis de ML de los dos conjuntos de datos fueron casi idénticas (Figura 1) y todas sus relaciones fueron respaldadas estadísticamente (es decir, valores de apoyo ≥ 75), excepto aquellas entre las muestras de *A. morenica*. Las hipótesis recuperaron la misma topología entre los ocho grupos de *Abronia* previamente definidos [38]. Sin embargo, las muestras de la presunta nueva especie, *A. morenica* y *A. ornelasi* comprendían un nuevo clado dentro del árbol *Abronia* que era el grupo hermano del grupo VII [38]. Las muestras de la presunta nueva especie fueron respaldadas significativamente como el taxón hermano de *A. morenica*. Por lo tanto, la presunta nueva especie está relacionada solo lejanamente con *A. lythrochila* (grupo VII, subgénero *Auriculabronia*), y está aún más distantemente relacionada con *A.* sp. "Laguna Bélgica" (grupo VI, subgénero *Scopaeabronia*), que son las *Abronia* arborícolas geográficamente más cercanas. Asignamos tentativamente la presunta nueva especie al subgénero *Lissabronia*, pero presentamos arreglos taxonómicos alternativos en la sección de Discusión.

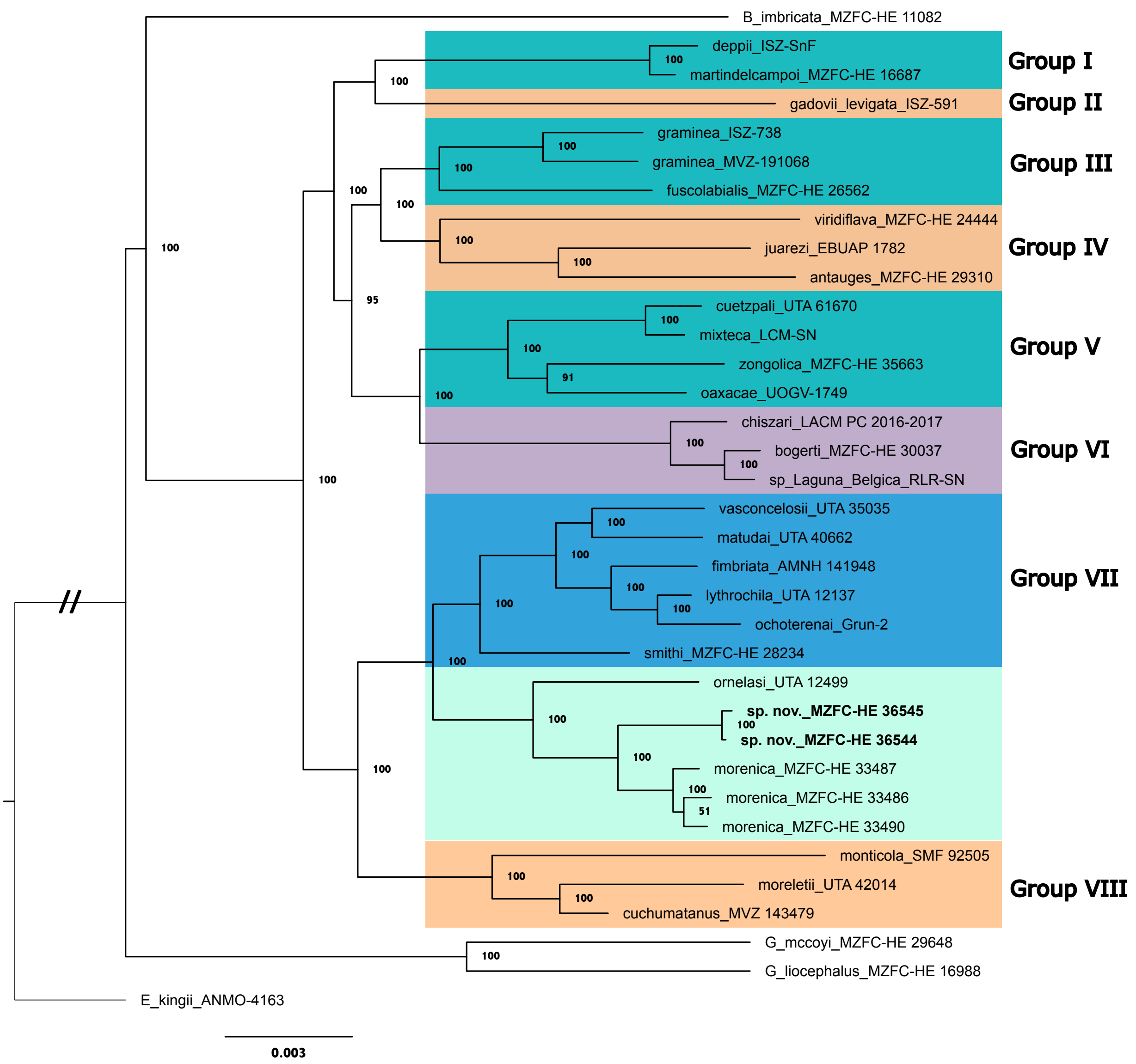

Fig 1.

Hipótesis filogenética de máxima verosimilitud para el género *Abronia* basada en 3425 loci RADseq. Los números en las ramas son valores de arranque. B. = *Barisia*; E. = *Elgaria*; G. = *Gerrhonotus*. Los grupos de especies son aquellos en la hipótesis filogenética molecular más reciente de *Abronia* [38]. Los nombres de los grupos de especies (I-VIII) siguen la hipótesis filogenética molecular más reciente de *Abronia* [38]. Los cuadros de color indican asignaciones taxonómicas basadas en morfología [39]: subgénero *Abronia* (turquesa), subgénero *Scopaeabronia* (violeta), subgénero *Auriculabronia* (azul), subgénero *Abaculabronia* más *Lissabronia* (verde pálido) y anterior género *Mesaspis* (naranja pálido).

Aunque no es concluyente con respecto al carácter distintivo de la presunta nueva especie de *A. morenica*, las distancias genéticas por pares no corregidas (distancias p) son informativas. La distancia p media entre muestras de la presunta nueva especie y entre muestras de *A. morenica* fue de 0.000515 y 0.001123, respectivamente. Por el contrario, la distancia p media entre la presunta nueva especie y *A. morenica* fue de 0.004032. Este valor supera las distancias p

medias entre otras especies hermanas de *Abronia* que se reconocen como taxones distintos (*A. deppii*/*A. martindelcampoi*, distancia p = 0.002076; *A. cuetzpali*/*A.mixteca*, 0.002684; *A. bogerti*/*A. chiszari*, 0.003464), pero es menor que otros pares de especies dentro del género (p. ej., *A. oaxacae*/*A. zongolica*, 0.008179).

El análisis de validación cruzada de conStruct de la presunta nueva especie, *A. morenica* y *A. ornelasi* indicó que el mejor modelo (es decir, el más simple con mejor precisión predictiva que otros) era un modelo con K = 3; sin embargo, la precisión predictiva de los modelos espacial y no espacial en K = 3 no difería sustancialmente entre sí (Figura 2). Esto sugiere que el aislamiento por distancia no es una característica de los datos. Además, los análisis con el modelo no espacial y espacial y K = 3 mostraron que las muestras de la presunta nueva especie y las muestras de *A. morenica* extraen la mayor parte de su ascendencia de la misma capa (Figura 3). Sin embargo, las primeras muestras de la presunta nueva especie también extraen alrededor de un tercio de su ascendencia de una segunda capa, mientras que la muestra de *A. ornelasi* extrae toda su ascendencia de una tercera capa. Estos resultados son consistentes con un ancestro común de la presunta nueva especie y *A. morenica*, y sugieren una divergencia genética moderada luego de la separación geográfica presumiblemente reciente de estos dos linajes.

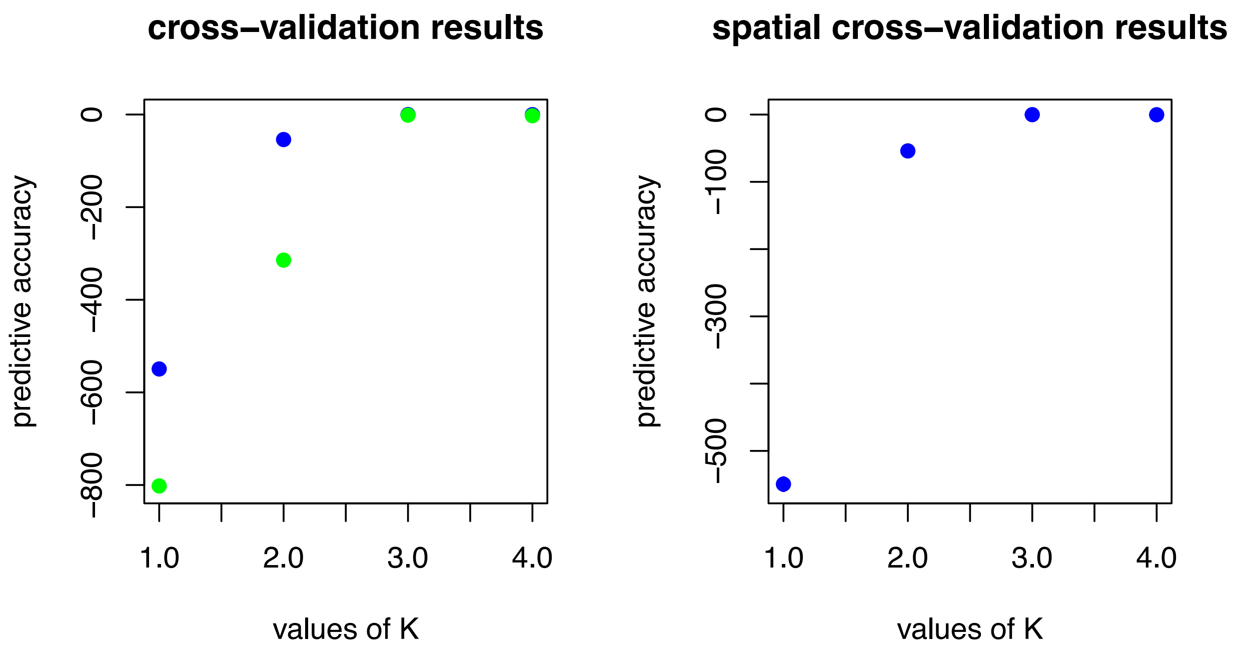

Fig 2.
Resultados de validación cruzada del análisis de conStruct que compara los modelos de conStruct espaciales y no espaciales (en azul y verde, respectivamente) ejecutados con K 1 a 4.

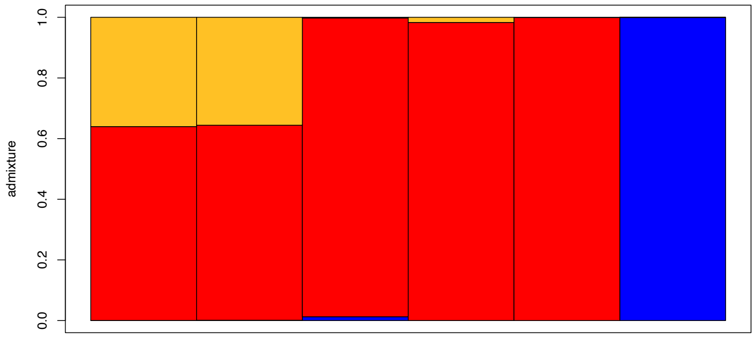

Fig 3.

Resultados del análisis de conStruct usando K = 3 para el modelo espacial que muestra las contribuciones de las capas. Muestras de izquierda a derecha: presunta nueva especie (dos primeras muestras), *A. morenica* (tres muestras siguientes) y *A. ornelasi*.

La media de gdi y el intervalo de confianza del 95 % (entre paréntesis) estimados para la presunta nueva especie *A. morenica* y *A. ornelasi* fueron 0.8093 (0.8084–0.8101), 0.3857 (0.3847–0.3868) y 0.9246 (0.9239–0.9252), respectivamente (Figura 4). Se ha sugerido como regla general que los valores de gdi < 0.2 sugieren una sola especie y los valores de gdi > 0.7 sugieren especies distintas, mientras que los valores de gdi dentro del rango indican una delimitación ambigua [70]. Bajo este marco de interpretación, nuestro análisis BPP es ambiguo con respecto a si la presunta nueva especie justifica el reconocimiento como un taxón distinto de *A. morenica*, lo cual es congruente con el escenario de divergencia reciente sugerido por el análisis conStruct (ver arriba).

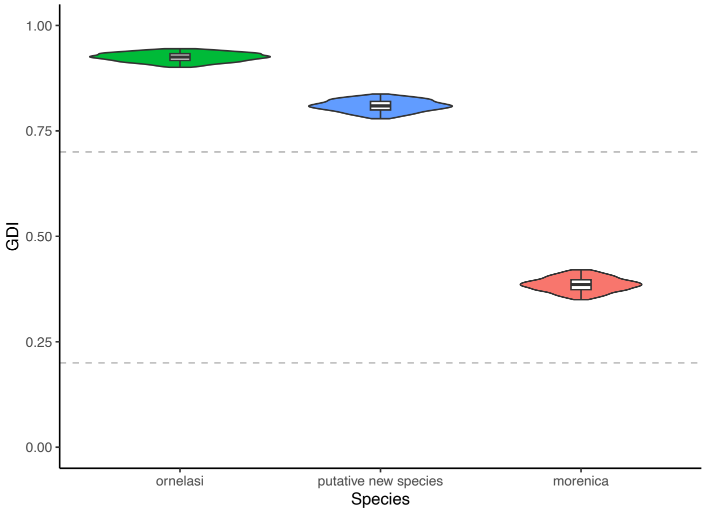

Fig 4.

Resultados de la delimitación de especies en el clado (*A. ornelasi* (*A. morenica*, presunta nueva especie)) aplicando el índice heurístico gdi a estimaciones de parámetros de BPP. Las líneas de puntos corresponden a gdi = 0,2 y 0,7. Las líneas horizontales representan medias y los cuadros intervalos de confianza del 95% alrededor de las medias.

La evidencia morfológica es congruente con la evidencia genómica de que la presunta nueva especie y *A. morenica* están estrechamente relacionadas, y también congruente con nuestra interpretación de que son taxones distintos. Los cinco especímenes de la presunta nueva especie son asignables al subgénero *Lissabronia*, al que esta asignado *A. morenica* [44], porque todos tienen 7 de los 10 caracteres diagnósticos de ese clado [1] y porque no tienen las sinapomorfías diagnósticas de cualquiera de los otros subgéneros reconocidos (*Lissabronia* únicamente carece de sinapomorfías) [1, 39]. Múltiples características morfológicas distinguen fácilmente a la presunta nueva especie de *A. morenica* y de todas las demás especies de *Lissabronia* (ver la sección de Comparaciones, a continuación). Corroborando esta evidencia morfológica, la presunta nueva especie está aislada geográficamente de todas las poblaciones conocidas de los miembros del género tanto arborícolas como terrestres. Dentro de *Lissabronia*, la población conocida más cercana es la de *A. morenica* de la localidad tipo en la parte occidental de la Sierra Madre de Chiapas, unos 110 km al sur-suroeste [44]. La brecha geográfica entre la presunta nueva especie y *A. morenica* se extiende por las tierras bajas de la Depresión Central de Chiapas. Esta amplia depresión semiárida es ampliamente reconocida como una barrera que impide la dispersión para las ranas [18], salamandras [81], serpientes [26, 82, 83] y lagartijas [27] que habitan en las montañas, con diferentes especies del mismo género a ambos lados de la Depresión. Con base en esta evidencia genómica, morfológica y biogeográfica concordante, consideramos que los especímenes de *Abronia* de Coapilla representan una especie no descrita, para la cual proponemos el nombre:

Abronia cunemica Clause, Luna-Reyes, Mendoza-Velázquez, Nieto-Montes de Oca & Solano-Zavaleta **sp. nov.**
urn:lsid:zoobank.org:act:5E56D78F-1DEF-42B3-AEBD-B2AD2E1D1817
Dragoncito de Coapilla (nombre común recomendado en español)
Coapilla Arboreal Alligator Lizard (nombre común recomendado en inglés)
Figuras 5–8, Cuadro 1

Holotipo (Figuras 5–8, Cuadro 1)

MZFC-HE 36544 (número de serie de campo AGC 1428), macho adulto, alrededores de Coapilla, Municipio de Coapilla, Montañas del Norte, Chiapas, México (17.14°, -93.16°, datum WGS 84), 1625 m de elevación, Adam G. Clause, Emmanuel Javier-Vázquez, Ana Reyna Pale Morales, 14 de agosto de 2021.

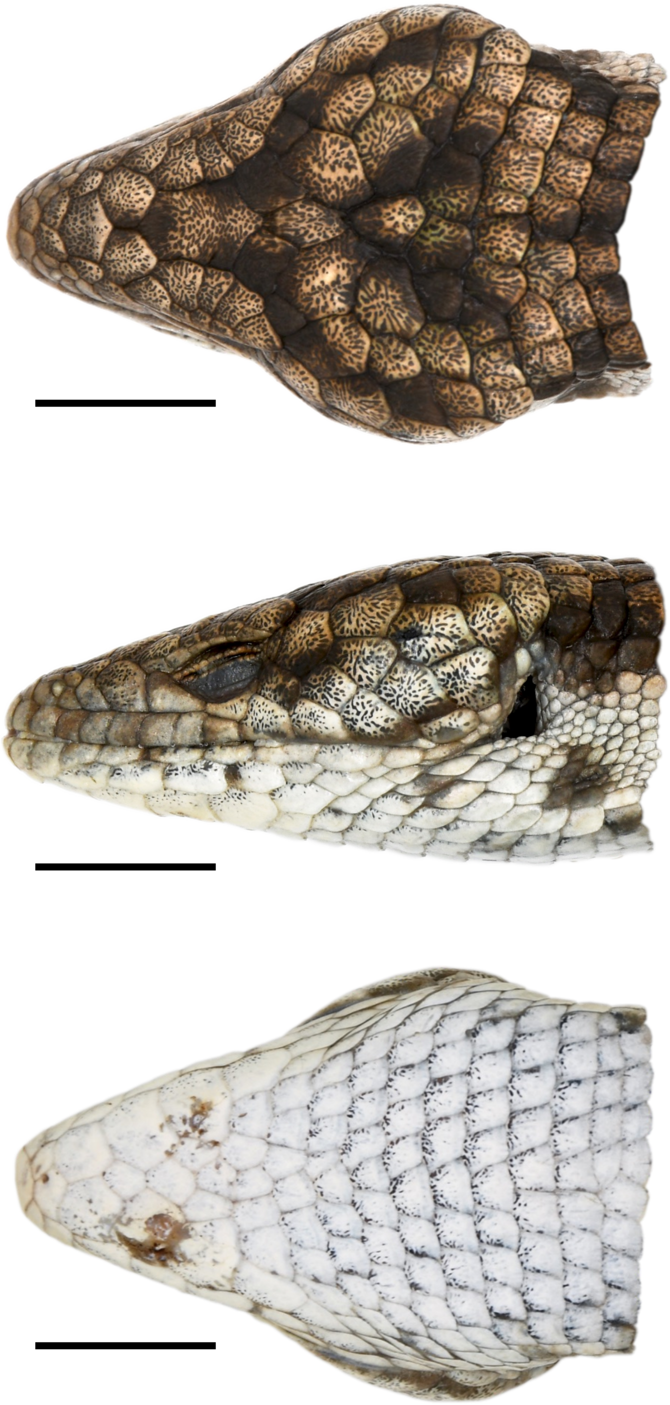

Fig 5.

Holotipo de *Abronia cunemica* sp. nov. de Coapilla, Chiapas, México (MZFC-HE 36544, 29 mm de longitud de la cabeza). Vista dorsal (superior), vista lateral izquierda (media) y vista ventral (inferior) de la cabeza en preservación. Todas las barras de escala = 10 mm. Fotografías por Israel Solano-Zavaleta.

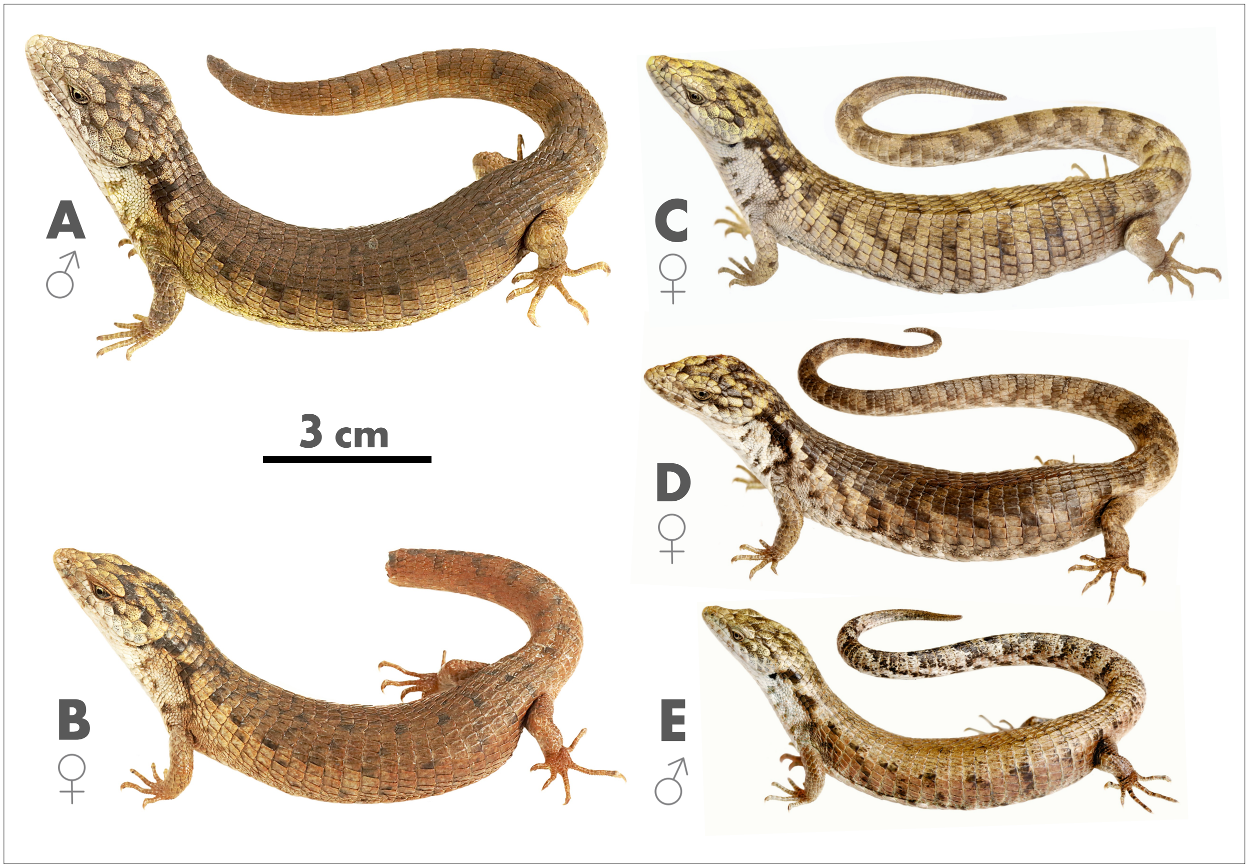

Fig 6.
Variación de color en vida de la serie tipo de *Abronia cunemica* sp. nov. de Coapilla, Chiapas, México. (A) Macho adulto holotipo, MZFC-HE 36544, 127 mm de longitud hocico-cloaca (LHC); (B) hembra adulta paratipo, MZFC-HE 36545, 113 mm de LHC; (C) hembra adulta paratipo, MZFZ 4514 (AGC 1492), 110 mm de LHC; (D) hembra adulta paratipo, MZFZ 4513 (AGC 1491), 107 mm de LHC; (E) macho juvenil paratipo, MZFZ 4512 (AGC 1484), 91 mm de LHC. Fotografías por Emmanuel Javier-Vázquez.

Paratipos (Figuras 6–7, Cuadro 1, n = 4)

MZFC-HE 36545 (número de serie de campo AGC 1429), hembra adulta, todos los datos de colecta son los mismos del holotipo. MZFZ 4512 (AGC 1484), macho juvenil, alrededores de Coapilla, Municipio de Coapilla, Montañas del Norte, Chiapas, México (17.13°, -93.16°, datum WGS 84), 1605 m de elevación, Oscar M. Mendoza-Velázquez, Candelario Cundapí-Pérez, Roberto Luna-Reyes, Adam G. Clause, Marcos Joaquín Fitz-Pérez, José Manuel Toledo-Morales, Emmanuel Javier-Vázquez, Daniel Lara-Tufiño, 16 de febrero de 2022. MZFZ 4513–4514 (AGC 1491–1492), dos hembras adultas, todos los datos de colecta son los mismos del MZFZ 4512 excepto que fueron colectadas el 18 y 19 de febrero de 2022, respectivamente. Las coordenadas de GPS en este párrafo y el párrafo anterior son imprecisas intencionalmente; los detalles están disponibles en la subsección de Conservación.

Diagnosis

Abronia cunemica sp. nov. se puede distinguir de todos los congéneres reconocidos, incluidas todas las especies anteriormente consideradas como miembros del género *Mesaspis*, por la siguiente combinación de caracteres: (1) la ausencia de escamas supra-auriculares protuberantes o similares a espinas; (2) la ausencia de escamas posterolaterales de la cabeza protuberantes o en forma de casco; (3) dorso de la cabeza amarillo pálido con marcas oscuras distintivas; (4) 35–39 hileras transversales de escamas dorsales; (5) escamas de la primera hilera de ventrales más grandes en relación con el tamaño de las escamas de la hilera medial adyacente; y (6) dorso marrón con bandas transversales oscuras, que a veces se reducen a una serie de manchas oscuras.

Comparaciones

Abronia cunemica sp. nov. se puede diferenciar de todas las especies del anterior género *Mesaspis* por tener 35–39 hileras de escamas dorsales transversales (vs. >40 hileras). Además, la nueva especie se diferencia de *A. cuchumatanus*, *A. gadovii* y el complejo de especies de *A. moreletii* por tener 14 hileras de escamas dorsales longitudinales (vs. 16 o 18, 16–18, y 18–22, respectivamente); de *A. antauges* y *A. juarezi* por tener escamas dorsales vertebrales y paravertebrales fuertemente quilladas en la mitad del cuerpo (vs. lisas a ligeramente convexas); de *A. viridiflava* por tener la escama frontonasal presente (vs. ausente); y de *A. monticola* por tener una escama postmentonal dividida o parcialmente dividida en 3/5 o 60% de los especímenes (vs. no dividida). Entre las especies de *Abronia* que no se consideraban miembros del anterior género *Mesaspis*, *Abronia cunemica* sp. nov. difiere de cada subgénero reconocido de la siguiente manera, con estado(s) de carácter para cada subgénero entre paréntesis y las sinapomorfías subgenéricas (si existen) indicadas en letra cursiva. A diferencia del subgénero *Scopaeabronia*, la nueva especie tiene la escama temporal primaria inferior sin expandirse (vs. *expandida*), 6 hileras longitudinales de escamas nucales (vs. *8 hileras*) y de 35–39 hileras transversales de escamas dorsales (vs. *38–47*). A diferencia del subgénero *Auriculabronia*, la nueva especie carece de escamas supra-auriculares fuertemente protuberantes o similares a espinas (vs. *presentes*). Dentro del subgénero *Auriculabronia*, la nueva especie se diferencia aún más de *A. matudai* (una especie en la que las escamas supra-auriculares a veces difícilmente sobresalen) por tener una barra lateral oscura en el cuello, aunque a menudo dividida en dos manchas separadas (vs. ausente); y por tener un dorso marrón con bandas transversales oscuras, aunque a menudo reducidas a una serie de manchas (vs. dorso verde sin bandas transversales en los machos adultos). A diferencia del subgénero *Abronia*, la nueva especie tiene la hilera más lateral de escamas ventrales claramente agrandada en relación con la hilera media adyacente (vs. no agrandada). Dentro del subgénero *Abronia*, la nueva especie difiere aún más del grupo *deppii* (*A. cuetzpali*, *A. deppii*, *A. martindelcampoi*, *A. mixteca* y *A. oaxacae* según lo definido por Campbell et al. [84], contra Campbell y Frost [39]) por tener escamas dorsales en los flancos arregladas en hileras longitudinales paralelas en relación con el pliegue ventrolateral (vs. *hileras longitudinales oblicuas*), y de *A. zongolica* por tener de 35–39 hileras transversales de escamas

dorsales (vs. 30–34). A diferencia del subgénero *Aenigmabronia*, la nueva especie tiene una escama occipital (vs. *dos occipitales*) y dos hileras de escamas que separan la escama occipital de la primera hilera transversal de escamas nucales (vs. *una hilera de escamas que separa las occipitales de las nucales*). A diferencia del subgénero *Abaculabronia*, la nueva especie en vida tiene un dorso marrón con al menos rastros de bandas transversales en adultos (vs. *en vida dorso verde oliva con márgenes de escamas pálidas o amarillas y sin rastro de bandas transversales en adultos*), y escamas supranasales sin contacto en todos especímenes (vs. *supranasales en contacto en 8/9 u 89% de los especímenes*). Dentro del subgénero *Lissabronia*, no hay un carácter único que permita diferenciar la nueva especie de los cuatro miembros actualmente reconocidos del grupo. Sin embargo, se puede distinguir de todas las especies de *Lissabronia* excepto de *A. morenica* por tener de 35–39 hileras transversales de escamas dorsales (vs. 28–32). A diferencia de *A. morenica*, los adultos de la nueva especie alcanzan una longitud hocico-cloaca de 107–127 mm (vs. 92–93 mm), y el dorso de la cabeza es de color amarillo pálido con distinguidas marcas oscuras (vs. gris pálido o bronceado con marcas oscuras ausentes o tenues). El Cuadro 1 presenta caracteres adicionales que diferencian a las especies de *Lissabronia* de *A. cunemica* sp. nov. y/o entre cada una de ellas.

Cuadro 1.
Características seleccionadas de *Abronia cunemica* sp. nov. y todas las especies en el clado congenérico *Lissabronia*.

Carácter	*A. cunemica* sp. nov.	*A. frosti*	*A. montecristoi*	*A. morenica*	*A. salvadorensis*
Coloración dorsal del cuerpo de adultos en vida	Marrón con bandas transversales oscuras (a veces indistintas)	Negro o marrón negruzco con marcas transversales blancas o amarillas	Marrón, a veces con bandas transversales claras	Marrón con bandas transversales oscuras (a veces indistintas)	Marrón claro con bandas transversales oscuras
Coloración dorsal de la cabeza de adultos en vida	Amarillo pálido con marcas oscuras prominentes	Negro con marcas blanquecinas, amarillentas, o gris pálido	Marrón o gris sin marcas oscuras	Gris pálido o bronceado con marcas oscuras indistintas o ausentes	Marrón o gris con marcas oscuras presentes o ausentes
Barra lateral oscura en el cuello entre el hombro y la abertura auricular	Presente (a menudo se divide en dos manchas)	Ausente	Ausente	Presente	Presente
Manchas de color amarillo o anaranjado en los flancos	Ausente (pero los flancos a veces son en su mayoría de color amarillo pálido)	Ausente	Ausente	Presente	Ausente
Escamas temporales primarias	4	2	4	4	4
Escamas occipitales*	1	1	3†	1 (86%) o 3 (14%)	1 (25%) o 3 (75%)††
Hileras transversales de escamas dorsales	35–39	28–32	30–31	30–35	29–32
Hileras longitudinales de escamas ventrales	12	14–16	12	12	12–14
Longitud hocico-cloaca (mm) de adultos	107–127	100–110	85–93†††	92–93	80–111

* = los porcentajes representan la proporción de especímenes disponibles con el número dado de escamas occipitales. † = el conteo sigue la interpretación de Campbell et al. [1]; todos los autores anteriores contaron cinco escamas. †† = el conteo sigue la interpretación de Campbell y Frost [39] y Clause et al. [44]. ††† = Hidalgo [120] da la longitud hocico-cloaca del espécimen más grande conocido (KU 184046) como 90 mm, pero aquí seguimos la medida informada por Campbell y Frost [39].

Tenemos incertidumbre sobre al subgénero al que pertenece *A. cunemica* sp. nov. No obstante, provisionalmente lo asignamos a *Lissabronia* basado en nuestro análisis filogenético (ver arriba). Además, a diferencia de todos los demás subgéneros, no hay un carácter único que permita distinguirla claramente de *Lissabronia* con base en el diagnóstico proporcionado por Campbell et al. [1] y con la sucesiva descripción de *A. morenica* [44] que provisionalmente asignó esa especie a *Lissabronia*. En la sección de Discusión, presentamos una explicación más detallada de nuestra asignación tentativa de la nueva especie.

Descripción del holotipo (Figuras 5–8, Cuadro 1)

Macho adulto con ambos hemipenes evertidos, peso 37.6 g, longitud hocico-cloaca (LHC) 127 mm, longitud de cabeza desde rostral hasta borde anterior superior de abertura de oído 29 mm, ancho de cabeza en punto más ancho 22 mm, relación ancho/largo de cabeza = 75.9%, cola rota y en regeneración, longitud de cola 121 mm, y 50 verticilos caudales (incluida porción en regeneración).

Supranasales 1/1, ninguna expandida medialmente; posnasales 2/2, superior más pequeña que inferior; un par de internasales anteriores y posteriores situadas entre rostral y frontonasal, internasal anterior derecha anormalmente dividida en una escama medial grande y una lateral pequeña; prefrontales >2 veces el tamaño de internasales posteriores, en amplio contacto entre sí medialmente; cantales 1/1, separando internasal posterior y prefrontal; loreales 1/1, en contacto con ambas posnasales; cantoloreales 1/1, apenas se extienden sobre el dorso del canthus rostralis, en contacto reducido con supraocular media anterior, en amplio contacto con cantal, prefrontal, y supralabiales; supraoculares medias 5/5; supraoculares laterales 3/3; superciliares 6/6, la más anterior en contacto con cantoloreal y menos de 1.5 veces la longitud de superciliar contigua; preoculares 1/1, izquierda fusionada con subocular anterior; suboculares 1/2, anterior a la izquierda aberrantemente fusionada con preocular; suboculares posteriores no contactan temporal primaria más inferior; postoculares 2/3; frontal grande, sin contacto con frontonasal, contacto reducido con interparietal; parietales sin contacto con supraoculares medias; una occipital grande, ligeramente más grande que interparietal; dos hileras transversales de escamas que separan occipital de primera hilera transversal de nucales; temporales primarias 4/4, solo las dos más inferiores en contacto con postoculares a cada lado, tercera fusionada aberrantemente con cuarta temporal secundaria a cada lado; temporales secundarias 4/4, cuarta fusionada aberrantemente con tercera temporal primaria en cada lado; temporales terciarias 4/5; supralabiales 11/10, antepenúltima más posterior para alcanzar órbita; infralabiales 8/8; postmentonal dividida; tres pares de escudos geniales agrandados posteriores a postmentonal dividida, más posteriores de tamaño inferior a escudos geniales adyacentes anteriores, >1.5 veces el tamaño de escamas posteriores; sublabiales 5/5, más anterior en contacto con segunda infralabial pero no con postmentonal.

Número mínimo de hileras longitudinales de escamas nucales 6; hileras transversales de escamas dorsales 36–38; hileras longitudinales de escamas dorsales 14, dispuestas en hileras horizontales paralelas en costados del cuerpo; ocho hileras longitudinales de escamas dorsales

medias fuertemente aquilladas, volviéndose lisas en costados; hileras transversales de escamas ventrales 37; hileras longitudinales de escamas ventrales 12; escamas de la primera hilera de ventrales más grandes en relación con el tamaño de las escamas de la hilera medial adyacente, pero solo en tercio medio del cuerpo; osteodermos aparecen moderadamente bien desarrollados en cabeza y nucales adyacentes; esquinas posterolaterales de cabeza no sobresalientes o en forma de casco; osteodermos aparecen débilmente desarrollados o ausentes en cuerpo y cola; escamas supra-auriculares granulares, no protuberantes ni en forma de espinas; escamas en la superficie del cuello entre nucales laterales agrandadas y escamas ventrolaterales agrandadas (en adelante, escamas laterales de cuello) 8–10, granulares; antebraquiales desde inserción de extremidad anterior hasta muñeca 12–13; pliegue ventrolateral entre abertura auricular y extremidad anterior ausente; pliegue ventrolateral posterior a extremidad anterior moderadamente bien desarrollado, con 3–6 hileras longitudinales indistintas de escamas pequeñas y gránulos en piel intersticial; laminillas subdigitales en cuartos dedos de pies 19/19.

Coloración de holotipo

En vida, cuerpo marrón ocre medio que se torna amarillo pálido en nucales laterales y amarillo limón opaco con motas marrones en flancos. Cuerpo con manchas distintivas de color marrón oscuro en flancos y manchas tenues marrón oscuro en mitad del dorso, aparentemente los remanentes de 10 bandas transversales indistintas. Manchas oscuras separadas por 3 escamas en parte media de dorso y 1.5–2.5 escamas en costados. Mayoría de escamas del pliegue ventrolateral amarillo limón opaco, pero algunas mayor o totalmente de color marrón rojizo debido a continuación ventrolateral de bandas dorsales. Escamas laterales del cuello mayormente blanquecino a amarillo limón opaco, con 3–4 manchas ventrolaterales marrón rojizo conspicuas. Escamas laterales del cuello bordeadas dorsalmente por una barra prominente, interrumpida o casi interrumpida negra a marrón oscuro que se extiende desde hombro hasta cerca del borde posterior superior de abertura auricular, 1–2 veces ancho de escamas nucales inferiores. Superficie dorsal de extremidades anteriores y posteriores de color marrón óxido, con algunos vetas amarillas y manchas marrón oscuro, más evidentes en extremidades anteriores. Dígitos de extremidades anteriores amarillo opaco con manchas marrón óxido, dígitos de extremidades posteriores marrón óxido. Cabeza amarillo pálido se torna de gris pálido a blanco lateralmente con manchas dorsales prominentes, vagamente en forma de punta de flecha, negras a marrón oscuro. Escamas de cabeza con rugosidad moderada acentuadas por manchas pequeñas y finas negras a marrón oscuro y vermiculaciones que se extienden a mayoría de nucales. Escamas dorsales entre narinas, y entre órbita y rostral en lados de cabeza (excepto en cantoloreal), sin manchas oscuras o vermiculaciones. Cola del mismo color que cuerpo, con 11 bandas transversales indistintas, pero mayormente ininterrumpidas de color marrón oscuro a negruzco que degradan ventralmente en un patrón similar a un tablero de ajedrez. Mandíbula inferior, barbilla y garganta blanco a gris pálido, muchas escamas con manchas oscuras distintivas en los márgenes anterior o dorsal, especialmente prominentes en sublabiales y escudos geniales. Porción anterior del vientre blanco a gris pálido hasta extremidades anteriores, tornándose

amarillo en resto del vientre, incluidas extremidades posteriores y base de cola, y beige con ligero tinte amarillo en resto de cola. Muchas escamas del vientre con marcas prominentes de color marrón ocre pálido, que se hacen más extensas lateralmente. Vientre de manos y patas anaranjado oxidado a amarillo oxidado. Iris amarillo pálido con abundantes manchas oscuras.

En preservación (etanol después de formalina), cuerpo marrón ocre medio con pequeñas porciones de algunas escamas que se tornaron verdosas, color crema en nucales laterales, y crema blancuzco hueso con motas marrones en flancos. Cuerpo con manchas marrones claras en flancos y manchas marrones débiles en el dorso. Mayoría de escamas en pliegue ventrolateral de color crema blancuzco hueso, pero algunas mayor o totalmente marrones debido a continuación ventrolateral de bandas transversales. Escamas laterales de cuello mayormente blanquecinas a crema blancuzco hueso, con 3–4 manchas ventrolaterales marrón verdoso conspicuas. Escamas laterales del cuello bordeadas dorsalmente por una barra prominente marrón oscura, interrumpida o casi interrumpida, que se extiende desde hombro hasta cerca del borde posterior superior de abertura auricular. Superficie dorsal de extremidades anteriores y posteriores de color oliva, con algunas vetas crema y manchas marrón claras, principalmente en extremidades anteriores. Dedos de manos crema blancuzco hueso a blanquecino con manchas marrón claro, dedos de pies marrón claro a oliva. Cabeza gris pálido que se torna blanca lateralmente con manchas dorsales marrones prominentes, vagamente en forma de punta de flecha. Escamas de cabeza con rugosidad moderada acentuadas por manchas pequeñas y finas marrón y vermiculaciones que se extienden a mayoría de nucales. Cola más clara que cuerpo, con bandas transversales mayormente de color marrón claro a marrón que ventralmente se desvanecen a color crema blancuzco hueso. Mandíbula inferior, barbilla, y garganta blanca a crema. Porción anterior del vientre hasta extremidades anteriores blanca, tornándose mayormente crema y crema blancuzco hueso lateralmente en el resto de vientre, incluidas las extremidades y la base de cola, y crema grisácea en resto de cola. Vientre de manos y patas azafrán.

Variación (Figuras 6–7)

Los cuatro paratipos son similares al holotipo en la mayoría de los aspectos, pero difieren en lo siguiente. Tres hembras adultas (MZFC-HE 36545 y MZFZ 4513–4514) con peso 22.1–24.3 g ($\bar{X}$ = 23.4), LHC 107–113 mm ($\bar{X}$ = 110), longitud de cabeza desde rostral hasta borde anterior superior de abertura de oído 22–23 mm ($\bar{X}$ = 22.3), ancho de cabeza en punto más ancho 16–17 mm ($\bar{X}$ = 16.3), relación ancho/largo de cabeza 72.7–73.9% ($\bar{X}$ = 73.1%), cola intacta y sin regenerar sólo en MZFC-HE 36545, con longitud de cola 156 mm (1.46 veces la LHC) y 97 verticilos caudales. Un macho juvenil (MZFZ 4512) con peso 13.9 g, LHC 91 mm, longitud de cabeza 18 mm, ancho de cabeza 12 mm, relación ancho/largo de cabeza 66.7%, cola rota y regenerándose, longitud de cola 126 mm (incluyendo tejido regenerado de 26 mm).

Supranasal izquierda expandida medialmente y desplazando internasal posterior adyacente en MZFC-HE 36545, y supranasal izquierda dañada en MZFZ 4514; escama ácigos medial presente entre internasales anteriores y posteriores en MZFZ 4514; prefrontales 2 veces el tamaño de internasales posteriores en MZFZ 4512, 2.5 veces el tamaño de internasales

posteriores en MZFC-HE 36545 y MZFZ 4513–4514, pero sin contacto entre sí en MZFC-HE 36545 y en reducido contacto entre sí en MZFZ 4513; cantoloreal sin (lado izquierdo) o apenas en contacto (lado derecho) con supraocular media anterior en MZFC-HE 36545, sin contacto con supraocular media en MZFZ 4513, y contacto muy estrecho en MZFZ 4514; superciliares 7/7 en MZFC-HE 36545 y MZFZ 4512–4513; superciliar más anterior casi 2 veces la longitud de superciliar contigua en MZFC-HE 36545 y MZFZ 4513, y casi la misma longitud que superciliar contigua en MZFZ 4512; suboculares 2/3 en MZFZ 4514, la parte anterior derecha dividida anormalmente en escamas posteriores pequeñas y anteriores grandes, y 2/2 en todos los demás paratipos; suboculares posteriores sin contacto con temporal primaria más inferior en todos los paratipos; postoculares 4/4 en MZFC-HE 36545 y MZFZ 4514, 3/3 en MZFZ 4512, y 3/4 en MZFZ 4513; contacto reducido entre frontal y frontonasal en MZFC-HE 36545, apenas contactando a frontonasal a través de un espolón delgado en MZFZ 4512 y MZFZ 4514; occipital más pequeña que interparietal en todos los paratipos, anormalmente dividida posteriormente en una pequeña escama semitriangular en lado izquierdo en MZFC-HE 36545 y en lado derecho en MZFZ 4513; dos hileras transversales de escamas separando occipital de nucales con la escama más posterior alargada que divide medialmente la primera hilera de nucales en MZFZ 4513; tercera temporal primaria aberrantemente fusionada con temporal secundaria superior en cada lado solo en MZFC-HE 36545; temporales secundarias 3/4 en MZFZ 4512–4513, segunda en lado izquierdo y tercera en lado derecho anormalmente agrandadas hacia atrás y parcialmente desplazando temporales terciarias adyacentes en MZFZ 4512, primera y segunda en lado izquierdo separadas por una segunda temporal terciaria anormalmente agrandada en MZFZ 4513, segunda en lado izquierdo anormalmente pequeña en MZFZ 4514; temporales terciarias 4–5 ($\bar{X}$ = 4.6), segunda anormalmente agrandada y desplazada anteriormente en MZFZ 4513; supralabiales 10–11 ($\bar{X}$ = 10.3); infralabiales 8–9 ($\bar{X}$ = 8.7), pequeña sublabial dividida de manera aberrante que casi separa octava y novena sublabiales del lado derecho en MZFZ 4514; postmentonal única en MZFZ 4513, parcialmente dividida posteriormente en MZFC-HE 36545 y anteriormente en MZFZ 4514; segunda sublabial en lado izquierdo dividida anormalmente en MZFZ 4513, quinta sublabial dividida de manera aberrante en ambos lados y escama más anterior contactando segunda (lado izquierdo) o tercera (lado derecho) infralabial en MZFZ 4514, sublabiales 4/5 y escama más anterior en contacto con tercera (lado izquierdo) o segunda (lado derecho) infralabial en MZFZ 4512.

Hileras transversales de escamas dorsales 35–39 ($\bar{X}$= 37.25); ocho hileras dorsales medias fuerte a moderadamente quilladas en MZFZ 4512 y MZFZ 4514; hileras transversales de escamas ventrales 37–40 ($\bar{X}$= 38.5); escamas laterales del cuello 8–9 en MZFC-HE 36545, 9–10 en MZFZ 4513–4514; laminillas subdigitales en cuartos dedos de pies 18–21 ($\bar{X}$= 19.6).

En vida, los tres paratipos hembras adultas (MZFC-HE 36545 y MZFZ 4513–4514) difieren del holotipo macho adulto de la siguiente manera: cuerpo marrón oscuro con tinte herrumbroso (MZFZ 4513) a marrón ocre pálido (MZFZ 4514); flancos marrón oxidado medio (MZFZ 4513) a marrón pálido (MZFZ 4514), tornándose gris pálido, beige, o crema anteriormente; restos de bandas transversales oscuras aún más reducidas en MZFC-HE 36545,

pero bandas transversales intactas, conspicuas, y aproximadamente en forma de V en MZFZ 4513–4514, separadas por 0.5–2 escamas en dorsales medias y 1.5–3.5 escamas lateralmente; escamas del pliegue ventrolateral de mismo color que escamas de flancos adyacentes, incluida continuación ventrolateral de bandas transversales más oscuras; escamas laterales del cuello blanquecinas a gris pálido, rara vez con tinte amarillo, y manchas ventrolaterales negruzcas a marrón medio; barra lateral oscura de cuello interrumpida en MZFC-HE 36545, completa en MZFZ 4513–4514; superficie dorsal de extremidades anteriores y posteriores blanquecina, gris pálido, marrón pálido, marrón medio, o marrón herrumbroso, a menudo con motas o manchas más oscuras y con extremidades anteriores siempre al menos parcialmente más pálidas que posteriores; dedos con varios tonos de gris o marrón, a menudo con manchas o marcas más oscuras, rara vez con motas o tintes de color crema; 4–5 manchas marrones alternas en escamas supralabiales y/o infralabiales en MZFC-HE 36545 y MZFZ 4513; escamas cantoloreales sin manchas oscuras y vermiculaciones; cola con 9 (MZFC-HE 36545, cola rota y sin regenerar), 14 (MZFZ 4514, cola en regeneración), o 22 (MZFZ 4513, cola intacta) bandas transversales marrón oscuro, algunas parcialmente fusionadas con bandas adyacentes; porción regenerada de cola en MZFZ 4514 casi completamente marrón oscuro; mandíbula inferior, barbilla y garganta sin manchas oscuras; vientre blanco a gris pálido que se torna gris más oscuro lateralmente, con franja ventral media de color amarillo pálido o crema posterior a extremidades anteriores; vientre de cola blanco a gris pálido con bandas transversales degradadas formando un patrón vago similar a un tablero de ajedrez de manchas gris más oscuras; vientre de manos y patas anaranjado oxidado brillante a marrón oxidado opaco, a veces tornándose amarillento basalmente; iris con ligero tinte verde en MZFC-HE 36545.

En vida, el paratipo macho juvenil (MZFZ 4512) difiere del holotipo macho adulto de la siguiente manera: cuerpo marrón pálido que se torna beige más pálido en flancos, con algunas manchas más oscuras en general y tinte crema tenue anteriormente; cuerpo con 11 bandas transversales poco conspicuas de color marrón oscuro, en gran parte reducidas a manchas en hileras de escamas vertebrales y paravertebrales, a menudo bordeadas posteriormente con motas o manchas gris pálido a beige, cada una separada por 2–3 escamas tanto en parte media de dorso como lateralmente; la mayoría de escamas en y a lo largo de pliegue ventrolateral de color beige, pero algunas mayormente de color marrón oscuro bordeadas posteriormente por manchas blancas o gris pálido debido a continuación ventrolateral de bandas transversales; escamas laterales del cuello blanquecinas, algunas con tinte crema y manchas ventrolaterales marrón medio; barra lateral oscura de cuello interrumpida; extremidades anteriores y dedos beige a marrón pálido con motas y manchas marrón medio; extremidades posteriores y dedos marrón medio con motas y manchas marrón oscuro; 3–4 manchas alternadas marrón pálido en escamas supralabiales; marcas amarillo pálido y marrón oscuro en dorso de cabeza descoloridas y menos contrastantes; escamas de cabeza lisas o débilmente rugosas, con manchas oscuras y vermiculaciones menos extensas; cola ligeramente más pálida que cuerpo, especialmente en parte posterior, pero bandas transversales oscuras más conspicuas que en cuerpo; parte regenerada de cola marrón pálido con manchas más oscuras y sin bandas transversales;

mandíbula inferior, barbilla y garganta sin manchas oscuras; porción anterior del vientre hasta extremidades anteriores blanco a gris pálido, tornándose amarillo pálido en resto de vientre incluyendo extremidades y base de cola, y beige a gris pálido en resto de cola; vientre de manos y patas amarillo anaranjado opaco.

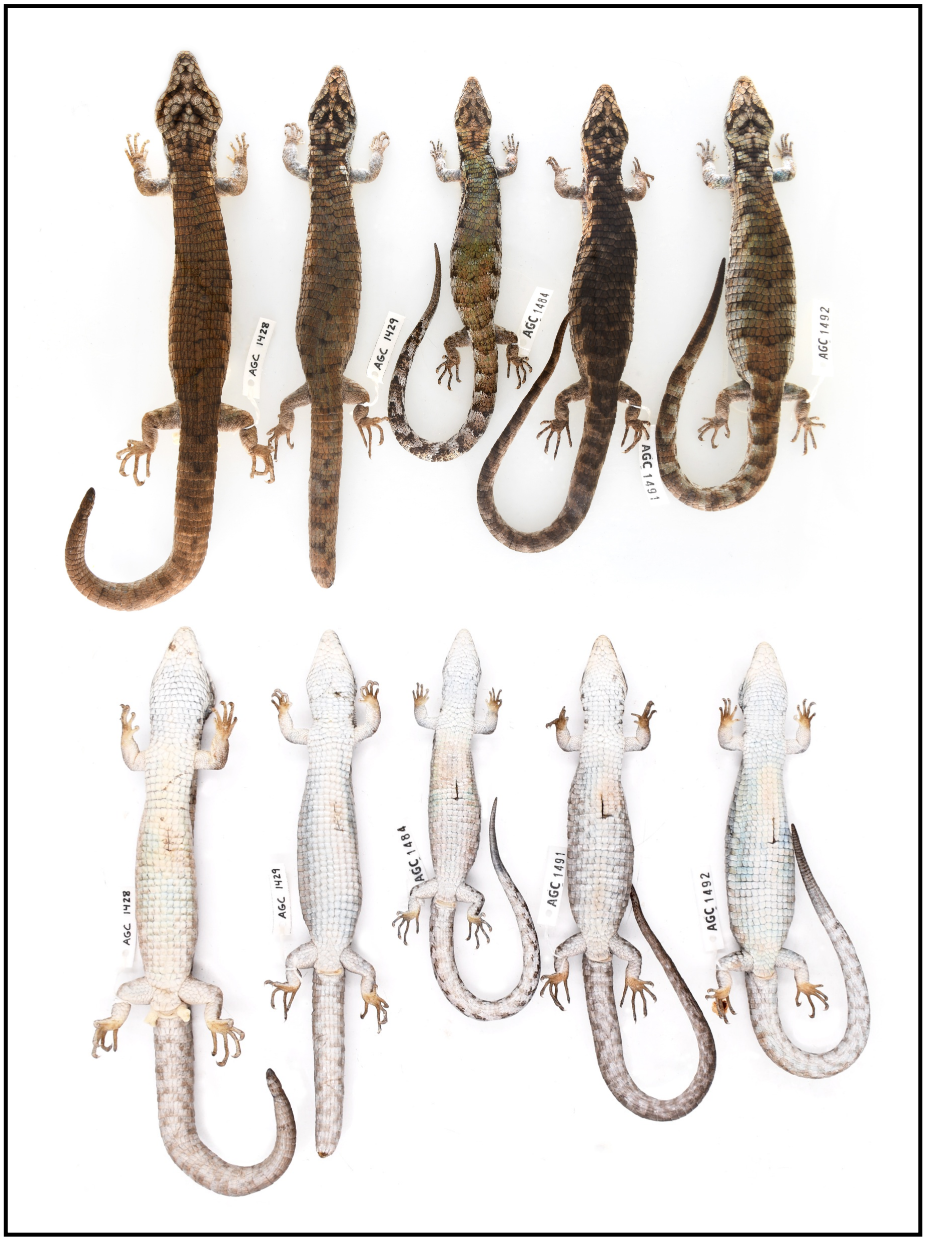

Fig 7.

Variación de color en preservación (etanol después de formol) en vista dorsal y ventral de la serie tipo de *Abronia cunemica* sp. nov. de Coapilla, Chiapas, México. De izquierda a derecha, macho adulto holotipo MZFC-HE 36544 (AGC 1428), 127 mm de longitud hocico-cloaca (LHC); hembra adulta paratipo, MZFC-HE 36545 (AGC 1429), 113 mm de LHC; macho juvenil paratipo, MZFZ 4512 (AGC 1484), 91 mm de LHC; hembra adulta paratipo, MZFZ 4513 (AGC 1491), 107 mm de LHC; y hembra adulta paratipo, MZFZ 4514 (AGC 1492), 110 mm de LHC. Fotografías por Israel Solano-Zavaleta.

En preservación (etanol después de formalina), todos los paratipos mantienen sus elementos básicos del patrón de color en vida, excepto que tonos marrón oxidado son reemplazados mayormente por varios tonos bronceado, crema, o gris pálido; dorso de cuerpo marrón oscuro a gris pálido o marrón pálido, pero escamas en paratipos de color más claro con tonos verdosos; bandas transversales dorsales oscuras ligeramente descoloridas y menos conspicuas; cuello generalmente más grisáceo, bronceado, o marrón pálido; dorso de la cabeza grisáceo; marcas en cabeza acentuadas y conspicuas en todos los especímenes; una marca oscura oblicua de tenue a conspicua se extiende desde margen posterior de frontal a temporales terciarias en todos especímenes; vientre de manos y patas amarillento a azafrán.

Etimología

El nombre de la especie es un adjetivo femenino singular en caso nominativo derivado de Cuñemo (ortografías alternativas: Kuñømø o Kujnyä'mä), que es el nombre de Coapilla en lengua indígena Zoque. Coapilla deriva de los términos náhuatl *coatl* (culebra) y *apan* (río) que significa "río de las culebras", mientras que Cuñemo se traduce de varias formas como "agua entre los árboles" [85], "lugar de la gran capital" [86], o "corona de cerros" según habitantes de la zona. El nombre elegido derivado de la lengua Zoque se refiere al ejido y municipio que alberga la única población conocida de la nueva especie. Nuestras consultas con los habitantes de Coapilla sobre este nombre recibieron respuestas universalmente positivas.

Distribución y ecología

Como la mayoría de las especies de dragoncitos arborícolas descritas en los últimos 30 años, *A. cunemica* sp. nov. solo se ha registrado en las cercanías de la localidad tipo (Figura 8). Sin embargo, al igual que muchos otros miembros del género, es probable que esta especie se encuentre más extensamente distribuida debido a la interconexión de bosques de tierras altas aparentemente adecuados. Todas las poblaciones documentadas se encuentran en una pequeña meseta boscosa. Al norte y al este, esta meseta se transforma en empinadas colinas que forman varios picos que superan los 2200 m de altitud y que dominan al pueblo de Tapalapa. Al sur y al oeste, la meseta termina bruscamente en un conjunto largo de dramáticos acantilados y promontorios que se ciernen sobre la ciudad de Copainalá. Las precipitaciones en esta meseta drenan hacia el Río Zacalapa al oeste o en el Río Chavarria al este. Ambas vías fluviales luego alimentan el Golfo de México, como parte de la cuenca del Río Grijalva.

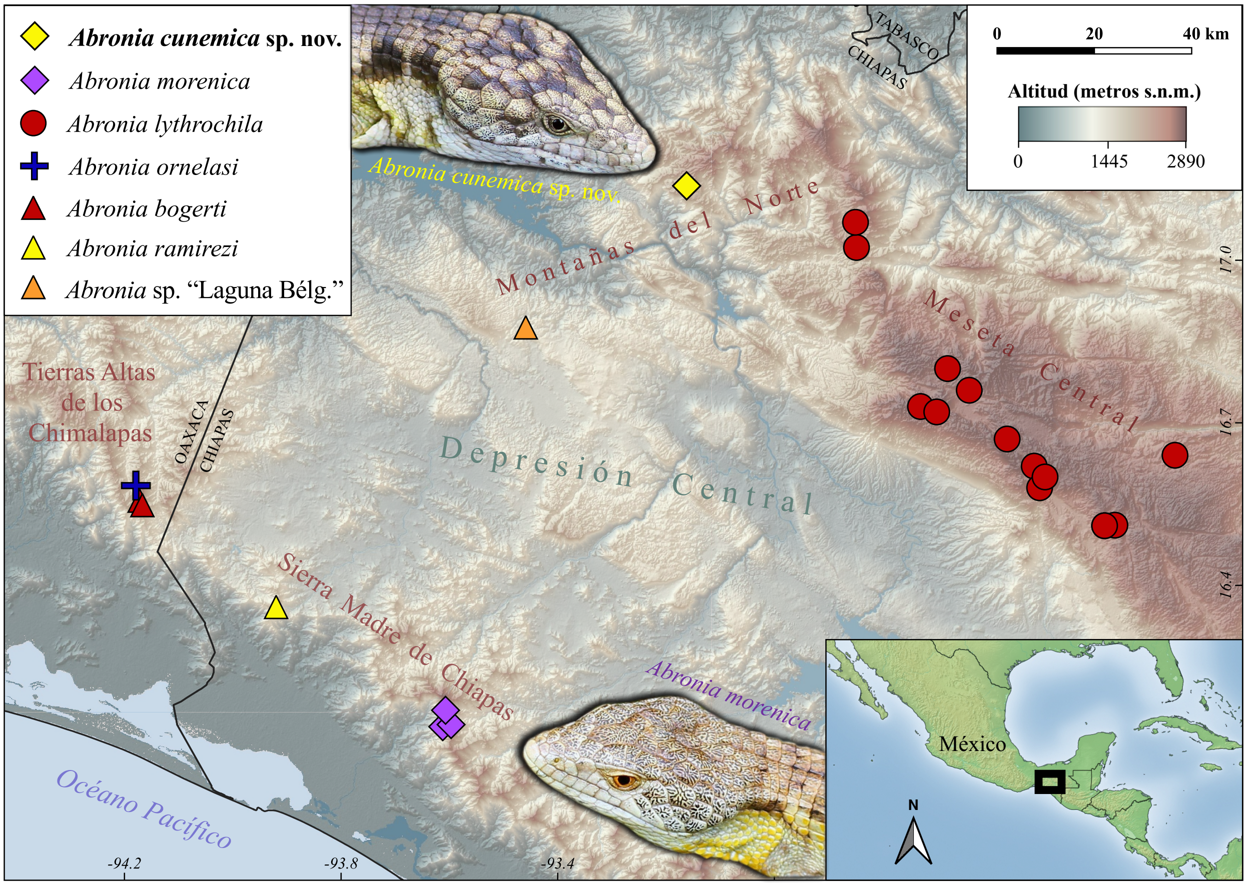

Fig 8.

Distribución geográfica de *Abronia cunemica* sp. nov. y congéneres arborícolas cercanos en Chiapas y Oaxaca, México. Las fotografías incluidas muestran las diferencias características en el color dorsal de la cabeza de adultos entre *A. cunemica* sp. nov. (amarillo pálido, con marcas oscuras conspicuas) y la estrechamente relacionada *A. morenica* (gris pálido/bronceado, con marcas oscuras ausentes o tenues). Se muestran los holotipos (machos adultos) de ambas especies; fotografía de *A. cunemica* sp. nov. intencionalmente reflejada horizontalmente. Fotografías por Adam G. Clause. Capas del mapa cortesía de Natural Earth (dominio público).

El bosque en y alrededor de la localidad tipo de *A. cunemica* sp. nov. es bosque de pino-encino, e incluye *Pinus chiapensis*, *P. maximinoi*, *Quercus crispipilis* y *Q. peduncularis* (Figura 9B). Aunque son poco comunes, los árboles *Carpinus caroliniana* y *Liquidambar styraciflua* también están presentes. El crecimiento epífito en el bosque ocupado es variable (Figura 9A, 9C). En algunos parches es extenso, dominado por masas densas de *Tillandsia fasciculata* y/o *T. rodrigueziana*. Otras bromelias menos abundantes incluyen *T. juncea*, *T. comitanensis* y/o *T. makoyana*, y una especie no identificada de *Catopsis*. Muchos árboles sostienen enredaderas de *Toxicodendron radicans*, que a veces forman grandes marañas que ascienden alto en el dosel. Los elementos menos conspicuos de la flora epífita incluyen al menos una especie de muérdago, algunas especies de helechos y varias orquídeas que incluyen *Domingoa purpurea* y una o más especies de los géneros *Epidendrum* o *Prosthechea*. El musgo epífito es generalmente escaso o

ausente, pero las ramas de muchos árboles están cargadas de líquenes crustosos, foliosos, y fruticosos de los géneros *Cladonia*, *Leptogium*, *Parmotrema*, *Pseudocyphellaria*, y *Usnea* [87].

En cinco viajes independientes a las cercanías de Coapilla (22–23 de agosto de 2015, 9–10 de agosto de 2021, 13–15 de agosto de 2021, 15–19 de febrero de 2022 y 5–7 de agosto de 2022), acumulamos más de 350 horas-persona de esfuerzo de búsqueda específica de *A. cunemica* sp. nov. en el hábitat ocupado, incluyendo la escalada con cuerdas en el dosel de casi 20 árboles para buscar entre las epífitas. A pesar de este esfuerzo, solo encontramos cinco individuos. Esta dificultad para encontrar a la especie se reflejó en nuestras conversaciones con los residentes locales. De aproximadamente dos docenas de personas que conocimos mientras caminábamos por senderos y caminos a través del bosque ocupado, solo la mitad de ellos reconocieron a la *Abronia* cuando se les mostró una fotografía, un video, o un animal vivo. Además, un estudiante que realizó un extenso trabajo herpetológico de campo en el Municipio de Coapilla, incluido un sitio que se superpone a la localidad tipo de *A. cunemica* sp. nov., no reportó observaciones de la especie [88].

El comportamiento arborícola de *A. cunemica* sp. nov., junto con el denso crecimiento de epífitas en muchos árboles de la zona, son probablemente los responsables de esta falta de familiaridad y pocas observaciones. Encontramos tres especímenes entre 3.5–19.5 m de altura en ramas de árboles vivos y muertos de *Pinus chiapensis*. También encontramos una pareja donde el macho sujeta a la hembra con una mordedura de cortejo en el suelo del bosque, después de que presumiblemente cayeran de un árbol adyacente de *Quercus* sp. Observamos individuos entre las 10:30–13:30 h, durante condiciones soleadas o parcialmente nubladas con temperaturas ambientales de entre 20–27 °C. Consistente con la temporada de apareamiento de verano/otoño documentada en otras especies de *Abronia* [89-91], encontramos la pareja de *A. cunemica* sp. nov. en cortejo el 14 de agosto. Aunque el macho inmediatamente dejó de morder a la hembra al momento de la captura, unas horas más tarde, cuando volvió a estar en contacto cercano de ella, volvió a morder y a sujetar de manera más persistente la cabeza y cuello de la hembra. Después de ser separados manualmente, unos minutos más tarde nuevamente pusimos a la pareja en contacto cercano. El macho respondió con episodios repetidos de temblores de cola sutiles pero intensos que duraron varios segundos. Dos hembras adultas recolectadas posteriormente a mediados de febrero parecían estar grávidas, aunque no diseccionamos los especímenes para confirmar esta sospecha.

El holotipo macho de *Abronia cunemica* sp nov. es inusualmente grande, con una LHC de 127 mm. Entre las especies arborícolas mexicanas del género, se informa que solo *A. mixteca* es más grande, con una LHC máxima conocida de 148 mm [39]. Las especies endémicas de Guatemala *A. anzuetoi* y *A. fimbriata* son las otras únicas especies que se sabe exceden de tamaño a *A. cunemica* sp. nov., alcanzando longitudes de 143 y 130 mm de LHC, respectivamente [39, 92]. Sin embargo, tenemos conocimiento de registros no publicados de *A. lythrochila* que superan los 127 mm LHC, esperamos que pronto estos datos sean publicados formalmente.

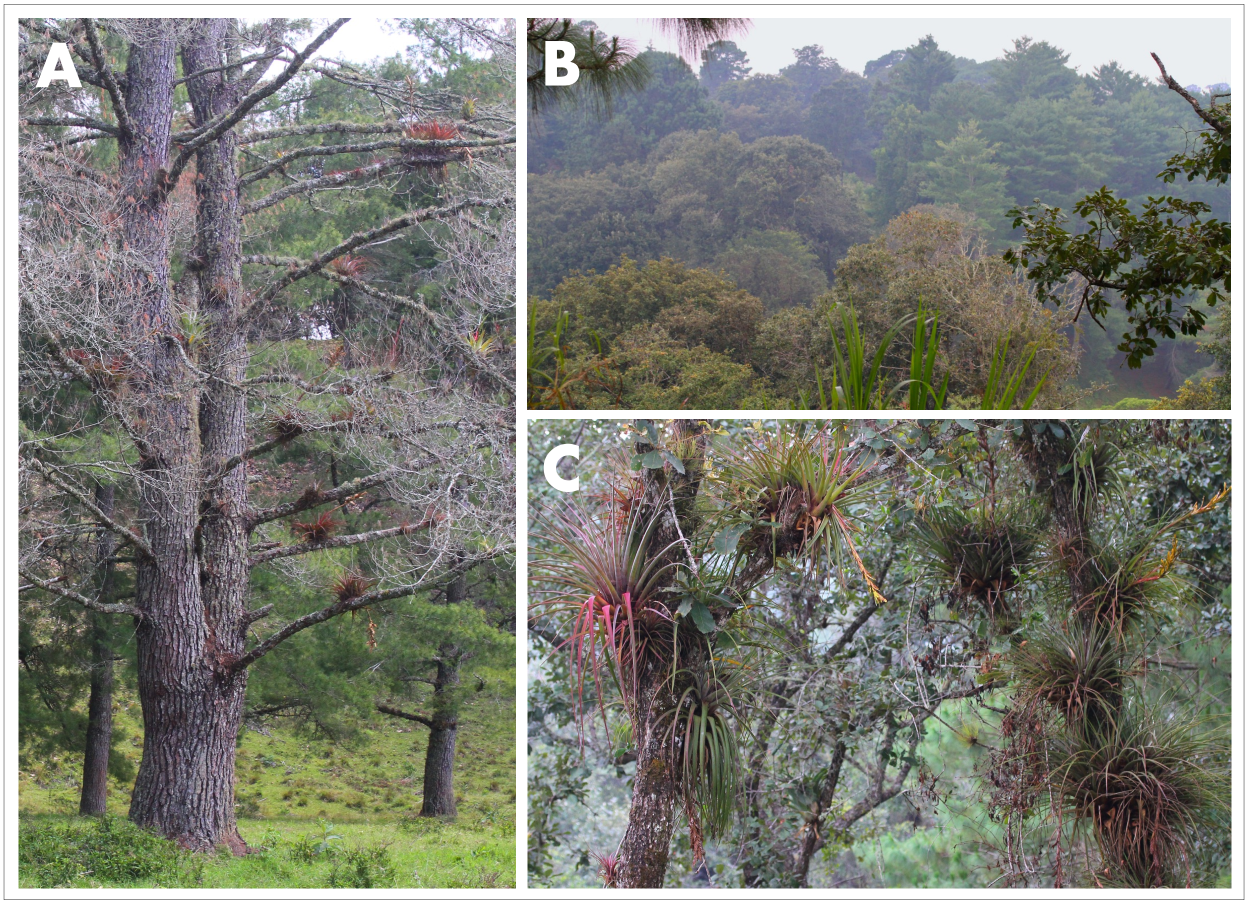

Fig 9.

Hábitat de *Abronia cunemica* sp. nov. cerca de Coapilla, Chiapas, México. (A) Árboles de *Pinus chiapensis* vivos y muertos en un potrero para la ganadería, (B) bosque intacto de *Pinus*-*Quercus*, (C) microhábitat de árboles de *Quercus* spp. cargados de epífitas. Fotografías tomadas por Adam G. Clause el 19 de febrero de 2022 (A) y el 14 de agosto de 2021 (B–C).

Sorprendentemente, un paratipo hembra adulta (MZFZ 4514) de *A. cunemica* sp. nov. fue reencontrada y recolectada 97 días después de que fue capturada, fotografiada, y liberada por OMMV el 14 de septiembre de 2021. Su ubicación cuando fue recapturada estaba a menos de 10 m de su ubicación de captura original, en un árbol de *Pinus chiapensis* adyacente al árbol de la misma especie donde fue encontrada originalmente. Aunque limitados, estos datos sugieren que *A. cunemica* sp. nov. puede ser relativamente sedentaria al menos algunos meses del año.

Basado en nuestro muestreo de campo, la herpetofauna arborícola o semiarborícola con la que *A. cunemica* sp. nov. ocurre en simpatría (dentro de 2 km de línea recta) se incluyen *Sceloporus internasalis* (MZFZ 4477), *Imantodes gemmistratus* (MZFZ 4562), *Bolitoglossa rufescens* (MZFZ 4473), *Bromeliohyla bromeliacia* (MZ-UNICACH 68–79) [93] y *Smilisca baudinii* (MZFZ 4561, 4565). Una o más de estas especies podrían ser una fuente de alimento para la nueva especie, y posiblemente *I. gemmistratus* podría comerse a las crías de *A. cunemica* sp. nov. Incluyendo registros anteriores [88], también documentamos la coocurrencia en simpatría de los siguientes reptiles: *Anolis tropidonotus* (=*A. spilorhipis* o *A. t. spilorhipis* de

algunos autores; MZFZ 4559–4560), *Scincella incerta* (MZFZ 4472), *Drymobius margaritiferus* (MZ-UNICACH 194) [88], *Geophis carinosus* (MZFZ 4475–4476), *Ninia diademata* (MZ-UNICACH 196, 376), *Pituophis lineaticollis* (MZ-UNICACH 198) [88], *Rhadinaea* [=*Rhadinella*] *godmani* (MZFC-HE 29997) [94], *Tantilla schistosa* MZFZ 4471, *Thamnophis cyrtopsis* (MZ-UNICACH 378) [88], *Micrurus elegans* (MZFZ 4474); y los siguientes anfibios: *Incilius valliceps* (MZ-UNICACH 95–98) [88], *Craugastor lineatus* (MZ-UNICACH 454) [88], *C. pygmaeus* (MZFZ 4563–4564) y *Rana brownorum* (MZ-UNICACH 155–157) [88]. Es probable que trabajo de campo posterior demuestre que *A. cunemica* sp. nov. coexiste con el congénere terrestre *Abronia temporalis*, que ha sido recolectado a menos de 8 km al noreste (MVZ 272336).

Conservación

Abronia cunemica sp. nov. no está registrada en ninguna de las áreas naturales protegidas. Sin embargo, varias parcelas de bosque dentro de un radio de 5 km de la localidad tipo han sido designadas como pequeñas reservas por los habitantes de Coapilla. Estos parches podrían albergar poblaciones de la especie. Al recorrer una de esas parcelas, encontramos un bosque con un dosel más intacto en comparación con otras tierras que visitamos, pero el sotobosque estaba muy modificado debido a la tala generalizada de árboles jóvenes (probablemente para leña). El Parque Ecoturístico Laguna Verde es otra área manejada por el Ejido de Coapilla donde probablemente *A. cunemica* sp. nov. se encuentre, y donde la comunidad promueve actividades de ecoturismo relativamente amigables con el medio ambiente. Sin embargo, problemáticas socioeconómicas que están bien documentadas en otras áreas protegidas de Chiapas también afectan a este parque ecoturístico y sus alrededores [95-98]. Por último, un área natural protegida adicional de 101 ha denominada Zona Sujeta a Conservación Ecológica Tzama Cum Pümy, que se encuentra a unos 7 km de la localidad tipo de *A. cunemica* sp. nov., también podría albergar la especie [99-101]. Son necesarias futuras encuestas, en estrecha colaboración con los líderes comunitarios de Coapilla y Tapalapa, para confirmar estas sospechas.

El bosque de la meseta donde *A. cunemica* sp. nov. se encuentra esta generalmente perturbado hasta cierto punto (p.ej., Figura 9A). Gran parte del paisaje se ha convertido en una sabana, con árboles muy alejados entre sí y un sotobosque cubierto de hierba y con pocos arbustos y árboles jóvenes. En repetidas ocasiones, también observamos camiones cargados con grandes troncos de pino recién cortados que salían del área. Dentro de los 100 m de todos los sitios donde encontramos *A. cunemica* sp. nov., vimos desarrollarse actividades agrícolas, ganaderas y forestales. Sin embargo, esta ausencia de bosques intactos y tala activa se ve atenuada por tres factores. Primero, muchas parcelas de propiedad comunal en el Ejido de Coapilla están bajo manejo forestal activo. Con base en la señalización pública que observamos, este enfoque de gestión parece rechazar los claros a favor de la tala selectiva de forma cíclica en un mosaico de parcelas seleccionadas. En segundo lugar, nuestros muestreos revelaron que las hembras adultas y juveniles de *A. cunemica* sp. nov. pueden vivir en los árboles remanentes que

crecen dentro de los pastizales. Por lo tanto, la especie podría tener una tolerancia sustancial a la perturbación del bosque, de manera similar a informes de algunas otras especies arbóreas de *Abronia* [102]. En tercer lugar, los individuos de *A. cunemica* sp. nov. parecen pasar la mayor parte de sus vidas en el dosel del bosque, donde están inherentemente protegidas de la influencia humana directa. Por estas razones, a pesar de la continua pérdida de bosques que evidentemente elimina el hábitat de esta especie y pone en peligro su supervivencia, mantenemos una reservada esperanza sobre su pronóstico a largo plazo.

Hay razones adicionales para el reservado optimismo sobre el futuro de *A. cunemica* sp. nov., a nuestro juicio. Debido a que la especie vive en porciones de altitud media en las Montañas del Norte, probablemente tiene espacio para expandirse hacia arriba a medida que el cambio climático empuja el bosque de pino-encino hacia la misma dirección [28, 30, pero ver 31]. Esto es diferente a su pariente cercano *A. morenica*, que parece vivir solo en el bosque mesófilo de las cimas de las montañas, por lo tanto, está más amenazada por la pérdida de hábitat provocada por el cambio climático [44]. Además, aunque nos encontramos con algunas personas que erróneamente temían a *A. cunemica* sp. nov., creyendo que es venenosa, la falta general de familiaridad con la especie por parte de los miembros de la comunidad sugiere que las poblaciones no están sujetas a una presión seria por la matanza injustificada. Por último, aunque los miembros arborícolas del género *Abronia* son cada vez más el objetivo del comercio internacional ilegal de mascotas [103-105], sospechamos que la increíble dificultad que experimentamos para encontrar esta especie reducirá la intensidad de esta amenaza. No obstante, para reducir aún más cualquier posible presión de caza furtiva, hemos enmascarado intencionalmente las localidades mencionadas en este artículo, (1) redondeando las coordenadas GPS a la centésima de grado más cercana, y (2) no reportamos la distancia o la dirección desde el pueblo más cercano [106].

A pesar de que todas las conclusiones con respecto a las amenazas se basan en datos limitados y, por lo tanto, representan conjeturas informadas, consideramos justificable categorizar provisionalmente a *A. cunemica* sp. nov. en tres listas de especies en riesgo. Para la Lista Roja de Especies Amenazadas de la Unión Internacional para la Conservación de la Naturaleza, recomendamos que *A. cunemica* sp. nov. sea asignada como En Peligro de Extinción (*Endangered*) (B1ab[iii,v]+2ab[iii,v]) [107]. En el cumplimiento de los criterios para ser clasificada como En Peligro de Extinción, consideramos que la especie tiene una extensión de ocurrencia y un área de ocupación de mucho menor de 5,000 km^2 y 500 km^2, respectivamente; ser conocida de menos de cinco lugares; y estar experimentando una disminución inferida en la extensión del hábitat, la calidad del hábitat, y la cantidad de individuos maduros, debido a la tala de árboles y la posible pérdida de individuos debido a la matanza selectiva y la caza furtiva [107]. De manera similar, calculamos que *A. cunemica* sp. nov. tiene un Puntaje de Vulnerabilidad Ambiental (EVS por sus siglas en inglés, ver Wilson et al. [108]) de 18 de 20, lo que ubica a la especie en la categoría de Alta Vulnerabilidad. Obtuvimos este puntaje total con respecto a los criterios evaluados sobre la distribución geográfica (6 puntos, por tener una distribución limitada a México en las inmediaciones de la localidad tipo), distribución ecológica

(8 puntos, por ser conocida actualmente de un solo tipo de vegetación), y el grado de persecución humana (4 puntos, por tener hábitos arborícolas, aunque las personas piensan que son dañinos y pueden ser asesinados al encontrarlos). Finalmente, proponemos que *A. cunemica* sp. nov. sea categorizada como Amenazada en la lista de la Norma Oficial Mexicana [109], debido a que la especie cumple con los Criterios A.(I)+B.(II)+C.(II)+D.(III) como lo define el Anexo Normativo I o Método de Evaluación del Riesgo de Extinción de las Especies Silvestres en México (MER) [110]. Esta es la única lista para especies en riesgo que es reconocida legalmente por el gobierno de México. Sugerimos esta categorización basada en que *A. cunemica* sp. nov. presenta las siguientes características: una distribución muy restringida (4 puntos) que abarca menos del 5% del territorio nacional de México; un hábitat intermedio o limitante (2 puntos) con respecto a los requerimientos para el desarrollo natural de la especie, debido a su restricción a los bosques de altitudes medias; una vulnerabilidad media (2 puntos) intrínseca a la biología de la especie, debido a su historia natural especializada que depende de los árboles, pero permite que la especie persista en bosques fragmentados; y un impacto medio (3 puntos) por parte de los humanos sobre la especie debido a la presión de los asentamientos humanos, la fragmentación moderada del hábitat y el uso, comercio y tráfico potencial de la especie. La puntuación total de 11 puntos justifica una asignación a la categoría de Amenazada [109].

Discusión

Las especies de vertebrados recién descubiertas tienden a tener distribuciones geográficas pequeñas, lo que generalmente coloca a esas especies en inminente riesgo de extinción debido a que el tamaño del área de distribución influye fuertemente en ese peligro [111, 112]. Estos patrones son particularmente evidentes en las lagartijas, para los cuales la tasa de descripción de nuevas especies se está acelerando rápidamente [113]. Nuestro descubrimiento de *A. cunemica* sp. nov., una lagartija micro endémica que está en peligro de extinción, es consistente con estas tendencias globales.

Con el reconocimiento de *A. cunemica* sp. nov., el número de especies descritas en el género se incrementa a 42, de las cuales 32 se consideran arborícolas [45]. Es la 28va especie de *Abronia* conocida para México [45, 114]. El número total de lagartijas nativas descritas (excluyendo el clado Serpentes) en Chiapas ahora son 94 [44, 51, 115-119]. De estas especies de lagartijas, 10 son endémicas del estado de Chiapas [44, 52].

La posición filogenética del clado compuesto por *A. cunemica* sp. nov., *A. morenica* y *A. ornelasi* dentro del clado oriental de *Abronia* (según lo definido por Gutiérrez-Rodríguez et al. [38]) es congruente con sus distribuciones geográficas al este del Istmo de Tehuantepec. La topología de nuestro árbol filogenético abre tres posibles arreglos taxonómicos de alto nivel para el clado de *A. cunemica* sp. nov./*A. morenica*/*A. ornelasi*. Primero, el clado podría incluirse dentro del Grupo VII como se definió previamente [38]. En segundo lugar, el clado podría reconocerse como un nuevo noveno grupo de *Abronia* (Grupo IX). En tercer lugar, el clado podría reconocerse como dos nuevos grupos de *Abronia*: el Grupo IX (que incluiría a *A. ornelasi* y tentativamente *A. reidi*, que en conjunto corresponden al subgénero *Abaculabronia* [39]), y el

Grupo X (que incluiría a *A. cunemica* sp. nov., *A. morenica*, y tentativamente *A. frosti*, *A. montecristoi* y *A. salvadorensis*, que juntas corresponden al subgénero *Lissabronia* [1, 44]). Debido a que no había muestras genéticas disponibles de *A. reidi* (especie tipo de *Abaculabronia*) ni de *A. salvadorensis* (especie tipo de *Lissabronia*) para incluirlas en nuestra filogenia molecular, no tenemos confianza para elegir entre estas tres opciones taxonómicas. Sin embargo, preferimos provisionalmente la tercera opción y, por lo tanto, asignamos *A. cunemica* sp. nov. al subgénero *Lissabronia*. Estamos a la espera de la disponibilidad de una filogenia molecular con un muestreo más completo de especies arborícolas de *Abronia* para probar esta asignación.

Con la adición provisional de *A. cunemica* sp. nov. el contenido de *Lissabronia* se incrementa a cinco especies, siendo las otras *A. frosti* [1] de Guatemala, *A. montecristoi* [120] de El Salvador y Honduras; *A. morenica* [44] de México, y *A. salvadorensis* [120] de Honduras (especie tipo). Dentro de *Lissabronia*, *A. cunemica* sp. nov. parece ser morfológicamente más similar a *A. morenica* y *A. salvadorensis* entre todas las *Abronia* reconocidas (Cuadro 1). Este gran parecido con *A. salvadorensis* es sorprendente porque está separada por más de 600 km de *A. cunemica* sp. nov., lo que las convierte en las más distantes geográficamente de toda *Lissabronia*. La nueva especie se puede distinguir fácilmente de *A. morenica*, que se encuentra a 110 km al sur y es la especie de *Lissabronia* geográficamente más cercana, de la siguiente manera (estado del carácter de *A. morenica* entre paréntesis): de 35–39 hileras transversales de escamas dorsales (vs. 30–35), dorso de la cabeza amarillo pálido con distintivas marcas oscuras (vs. nunca amarillas y marcas oscuras ausentes o tenues), flancos en ocasiones en su mayoría amarillo pálido, pero mancha posterior amarillo o anaranjada en las escamas de los flancos ausente (vs. presente), y longitud de hocico-cloaca del adulto de 107–127 mm (vs. 92–93 mm). Estas diferencias físicas respaldan nuestra conclusión de que las dos poblaciones conocidas de *A. cunemica* sp. nov. y *A. morenica* no son miembros de la misma especie. Acumulativamente, nuestros análisis filogenéticos, genéticos de poblaciones y de delimitación de especies corroboran este reconocimiento propuesto de *A. cunemica* sp. nov. como una especie distinta de *A. morenica*. Reconocemos, sin embargo, que estos dos taxones no son muy divergentes genéticamente. También consideramos una interpretación alternativa de los datos genéticos: que *A. cunemica* sp. nov. simplemente representa la segunda población conocida de *A. morenica*. Dado su dramático aislamiento biogeográfico, con la inhóspita y semiárida Depresión Central de Chiapas que casi seguramente impide el flujo de genes en curso (Figura 8), en nuestra opinión, estas dos poblaciones están en trayectorias evolutivas separadas. Por lo tanto, rechazamos esta interpretación alternativa y, en cambio, consideramos que la población de Coapilla representa una especie independiente.

Aprovechamos esta oportunidad para revisar algunos estados de carácter problemáticos para los paratipos de *A. morenica*, que inadvertidamente fueron mal evaluados en la descripción original de la variación de los paratipos [44]. Estos errores no afectan el diagnóstico de *A. morenica*, ni afectan las comparaciones con *A. cunemica* sp. nov. No obstante, en este estudio los corregimos para una mayor claridad y para asegurar una interpretación morfológica consistente

para estos taxones que aparentemente están estrechamente relacionados. Las puntuaciones de estado de carácter actualizadas son las siguientes: escamas temporales secundarias 3/3, con la primera y la segunda escama en el lado izquierdo (MZFC-HE 33488) o en ambos lados (MZFC-HE 33484) separadas por la segunda temporal terciaria desplazada anteriormente; temporales terciarias 4–5 ($\bar{X}$ = 4.6); supralabiales 9–11 ($\bar{X}$ = 9.8), siendo la antepenúltima la más posterior en alcanzar la órbita en MZFC-HE 33485, 33488 y 34400; infralabiales 7–10 ($\bar{X}$ = 8.3); sublabiales 4–6 ($\bar{X}$ = 4.6).

A diferencia de la mayoría de las especies de lagartijas y serpientes recientemente propuestas para su reconocimiento en México, pero similar a otros miembros recientemente anunciados de su género [44, 45], *A. cunemica* sp. nov. hasta entonces era completamente desconocido para la ciencia. El descubrimiento de *A. cunemica* sp. nov. en las Montañas del Norte de Chiapas fue particularmente inesperado debido a la reducida distancia que separa a Coapilla de las poblaciones conocidas de *A. lythrochila* en la misma región fisiográfica. Esta última especie, la cual es miembro del subgénero *Auriculabronia* y, por lo tanto, no es un pariente cercano de *A. cunemica* sp. nov., ocurre a menos de 40 km al este-sureste y cerca del pueblo de Jitotol [60-62] (Figura 8). Las laderas y las crestas intermedias del corredor Tapalapa–Rayón–Pueblo Nuevo Solistahuacán sustentan grandes extensiones de bosque húmedo que parecen aptas para miembros arborícolas del género *Abronia*. Sin duda, nuevos estudios de este corredor poco explorado reducirán aún más la distancia que separa a estas dos especies. No obstante, los valles de baja altitud en las cercanías de Rayón convergen para crear un corredor de 1500 m de altitud que divide perfectamente las montañas más altas al este y al oeste. Consideramos plausible que esta división represente una barrera física que impide el contacto entre *A. cunemica* sp. nov. y *A. lythrochila*, pero ver García-Padilla y Escalante-Pliego [121].

A pesar de ser accesible por un camino pavimentado, lo que hace que Coapilla esté a menos de 3 horas en automóvil desde la ciudad más grande de Chiapas (Tuxtla Gutiérrez), la biodiversidad de vertebrados en el área de Coapilla aún no se conoce por completo. Antes del trabajo aquí publicado, solo se disponía de dos estudios de la herpetofauna local [88, 93]. El conjunto de mamíferos locales también está poco investigado. Se han documentado un total de 71 especies y subespecies de mamíferos en el Municipio de Coapilla [122, 123]. En particular, esto incluye el primer y segundo registro de Chiapas para las especies de murciélagos *Nyctinomops macrotis* y *N. laticaudatus*, respectivamente [122]. Sorprendentemente, no tenemos conocimiento de ningún estudio publicado de aves en el área. Sin embargo, los reportes informales indican que al menos dos aves llamativas y en riesgo (*Pharomachrus mocinno* y *Setophaga chrysoparia*) migran estacionalmente para vivir en o cerca de Coapilla [124].

Basándose en la presencia de estos murciélagos raramente documentados y aves en riesgo, recomendamos que *A. cunemica* sp. nov. sea promovida como una especie bandera para la conservación de esta región. Como miembro microendémico de un grupo de lagartijas llamativas e icónicas, consideramos que su potencial como especie clave es fuerte. No obstante, también reconocemos la importancia de las actividades educativas en la región para ayudar a prevenir el tráfico ilegal de la especie. La necesidad de mayores esfuerzos de conservación en las

Montañas del Norte es clara. Aunque Chiapas alberga la mayor extensión de bosque mesófilo de montaña de cualquier estado de México (6,037 km^2), con alrededor de una cuarta parte de esos bosques actualmente protegidos [28], la mayoría de esas Áreas Naturales Protegidas se encuentran en la Sierra Madre de Chiapas. De hecho, solo un área natural protegida reconocida internacionalmente incluye tierras por encima de los 1500 m de altitud en las Montañas del Norte: la Zona Sujeta a Conservación Ecológica Tzama Cum Pümy de 101 hectáreas [99-101]. Las partes de altitud media a alta de esta región fisiográfica, que ha experimentado una deforestación sustancial [125], representan un gran vacío en la red existente de áreas naturales protegidas en Chiapas [126]. La creación de nuevas áreas naturales protegidas y el fortalecimiento de la inversión social en áreas que ya reciben algún grado de protección a nivel local es una necesidad urgente [125, 127]. Las asociaciones u organizaciones para generar la aceptación de la comunidad entre las partes interesadas locales deben ser parte de tales programas [125, 128, 129]. Dado que el cambio de uso de tierra es el principal impulsor de la pérdida de biodiversidad a nivel mundial [130], alentamos los esfuerzos para salvaguardar los bosques donde habita *A. cunemica* sp. nov. Dichos esfuerzos servirían de base para llamar la atención y fomentar la conservación, que ha sido relegada desde hace mucho tiempo, de la notable biodiversidad de las Montañas del Norte de Chiapas.

Agradecimientos

Agradecemos a José Manuel Aranda-Coello por compartir generosamente información relevante. Por su ayuda con el trabajo de campo, estamos en deuda con los miembros de nuestro equipo: Emmanuel Javier-Vázquez, Candelario Cundapí-Pérez, Marcos Joaquín Fitz-Pérez, Ana Reyna Pale Morales, José Manuel Toledo-Morales, Víctor Vásquez-Cruz, Maisie G. MacKnight, Jorge Arturo Hidalgo-García, Daniel Lara-Tufiño y Justin K. Clause. A Uri Omar García-Vázquez que brindó importante apoyo logístico, por lo cual estamos agradecidos. Nuestro agradecimiento a Francisco Hernández-Najarro y Oscar Farrera-Sarmiento por la identificación de las plantas, a Ernesto Velázquez-Velázquez por autorizarnos el acceso a la colección del MZ-UNICACH, a Martha Erika Hernández de la Cruz y a Gloria Pérez por la información sobre el nombre de Coapilla en Zoque, y a Marcy Kinsey por su ayuda con la interpretación de los colores. La recolección de datos moleculares para este estudio fue financiada por una subvención de la Dirección General de Asuntos del Personal Académico, Universidad Nacional Autónoma de México (PAPIIT no. IN218522) a ANMO. Todos los análisis de los datos moleculares se realizaron en el clúster informático de alto rendimiento Mana de la University of Hawái. Agradecemos a Jorge Gutiérrez-Rodríguez por generar datos ddRADseq para algunas de las muestras utilizadas en nuestros análisis.

Apéndice S2.
Información de localidad y números de acceso de GenBank para las muestras utilizadas en este estudio.

Referencias

1. Campbell JA, Sasa M, Acevedo M, Mendelson III JR. A new species of *Abronia* (Squamata: Anguidae) from the High Cuchumatanes of Guatemala. Herpetologica. 1998;54(2):221–34.
2. Flantua SGA, Payne D, Borregaard MK, Beierkuhnlein C, Steinbauer MJ, Dullinger S, et al. Snapshot isolation and isolation history challenge the analogy between mountains and islands used to understand endemism. Glob Ecol Biogeogr. 2020;29:1651–73. doi: 10.1111/geb.13155
3. Wilson LD, McCranie JR. The herpetofauna of the cloud forests of Honduras. Amphib Reptile Conserv. 2004;3(1):34–48. doi: 10.1514/journal.arc.0000013
4. Rzedowski J. Analsis preliminar de la flora vascular de los bosques mesofilos de montaña de Mexico. Acta Bot Mex. 1996;35:25–44.
5. Perrigo AL, Hoorn C, Antonelli A. Why mountains matter for biodiversity. J Biogeogr. 2020;47:315–25. doi: 10.1111/jbi.13731
6. Antonelli A, Kissling WD, Flantua SGA, Bermúdez MA, Mulch A, Muellner-Riehl AN, et al. Geological and climatic influences on mountain biodiversity. Nat Geosci. 2018;11:718–25. doi: 10.1038/s41561-018-0236-z
7. García-Sotelo UA, García-Vázquez UO, Espinosa D. Historical biogeography of the genus *Rhadinaea* (Squamata: Dipsadinae). Ecol Evol. 2021;11:12413–28. doi: 10.1002/ece3.7988
8. Rovito SM, Wake DB, Papenfuss TJ, Parra-Olea G, Muñoz-Alonso A, Vásquez-Almazán CR. Species formation and geographical range evolution in a genus of Central American cloud forest salamanders (*Dendrotriton*). J Biogeogr. 2012;39:1251–65. doi: 10.1111/j.1365-2699.2012.02696.x
9. Bryson Jr. RW, Linkem CW, Pavón-Vázquez CJ, Nieto-Montes de Oca A, Klicka J, McCormack JE. A phylogenomic perspective on the biogeography of skinks in the *Plestiodon brevirostris* group inferred from target enrichment of ultraconserved elements. J Biogeogr. 2017;44(9):2033–44. doi: 10.1111/jbi.12989
10. Ornelas JF, Sosa V, Solis DE, Daza JM, González C, Soltis PS, et al. Comparative phylogeographic analyses illustrate the complex evolutionary history of threatened cloud forests of northern Mesoamerica. PLoS One. 2013;8(2):e56283. doi: 10.1371/journal.pone.0056283
11. Caviedes-Solis IW, Leaché AD. Leapfrogging the Mexican highlands: influence of biogeographical and ecological factors on the diversification of highland species. Biol J Linn Soc Lond. 2018;123:767–81. doi: 10.1093/biolinnean/bly002
12. Castoe TA, Daza JM, Smith EN, Sasa MM, Kuch U, Campbell JA, et al. Comparative phylogeography of pitvipers suggests a consensus of ancient Middle American highland biogeography. J Biogeogr. 2009;36(1):88–103. doi: 10.1111/j.1365-2699.2008.01991.x
13. Gutiérrez-Rodríguez J, Nieto-Montes de Oca A, Ortego J, Zaldivar-Riverón A. Phylogenomics of arboreal alligator lizards shed light on the geographical diversification of cloud forest-adapted biotas. J Biogeogr. 2022;49:1862–76. doi: 10.1111/jbi.14461

14. Birdlife International. *Pharomachrus mocinno. The IUCN Red List of Threatened Species* 2016: e.T22682727A92958465 [cited 2022 Dec 12]. Available from: http://dx.doi.org/10.2305/IUCN.UK.2016-3.RLTS.T22682727A92958465.en
15. Anderle RF. The Horned Guan in México and Guatemala. Condor. 1967;69(2):93–109. doi: 10.2307/1366601
16. Mason AJ, Grazziotin FG, Zaher H, Lemmon AR, Lemmon EM, Parkinson CL. Reticulate evolution in Nuclear Middle America causes discordance in the phylogeny of Palm-pitvipers (Viperidae: *Bothriechis*). J Biogeogr. 2019;46(5):833–44. doi: 10.1111/jbi.13542
17. Bogert CM. A new genus and species of dwarf boa from southern Mexico. Am Mus Novit. 1968;2354:1–38.
18. Duellman WE, Campbell JA. Hylid frogs of the genus *Plectrohyla*: systematics and phylogenetic relationships. Misc Publ Mus Zoology Univ Mich. 1992;181:1–32.
19. Caviedes-Solis IW, Nieto-Montes de Oca A. A multilocus phylogeny of the genus *Sarcohyla* (Anura: Hylidae), and an investigation of species boundaries using statistical species delimitation. Mol Phylogenet Evol. 2017;118:184–93. doi: 10.1016/j.ympev.2017.09.010
20. Sandoval-Comte A, Pineda E, Rovito SM, Luría-Manzano R. A new species of *Isthmura* (Caudata: Plethodontidae) from the montane cloud forest of central Veracruz, Mexico. Zootaxa. 2017;4277(4):573–82. doi: 10.11646/zootaxa.4277.4.7
21. Elias P. Salamanders of the Northwestern Highlands of Guatemala. Contr Sci Nat Hist Mus Los Angeles Co. 1984(348):1–20.
22. García-Castillo MG, Soto-Pozos ÁF, Aguilar-López JL, Pineda E, Parra-Olea G. Two new species of *Chiropterotriton* (Caudata: Plethodontidae) from Central Veracruz, Mexico. Amphib Reptile Conserv. 2018;12(2 [special section]):37–54 (e167).
23. Rovito SM, Vásquez-Almazán CR, Papenfuss TJ, Parra-Olea G, Wake DB. Biogeography and evolution of Central American cloud forest salamanders (Caudata: Plethodontidae: *Cryptotriton*), with the description of new species. Zool J Linn Soc. 2015;175:150–66. doi: 10.1111/zoj.12268
24. Jiménez-Arcos VH, Calzada-Arciniega RA, Alfaro-Juantorena LA, Vázquez-Reyes LD, Blair C, Parra-Olea G. A new species of *Charadrahyla* (Anura: Hylidae) from the cloud forest of western Oaxaca, Mexico. Zootaxa. 2019;4554(2):371–85. doi: 10.11646/zootaxa.4554.2.3
25. Arias E, Kubicki B. A new moss salamander, genus *Nototriton* (Caudata: Plethodontidae), from the Cordillera de Talamanca, in the Costa Rica-Panama border region. Zootaxa. 2018;4369(4):487–500. doi: 10.11646/zootaxa.4369.4.2
26. Campbell JA, Brodie Jr. ED. A new colubrid snake of the genus *Adelphicos* from Guatemala. Herpetologica. 1988;44(4):416–22.
27. Nieto-Montes de Oca A. The systematics of *Anolis hobartsmithi* (Squamata: Polychrotidae), another species of the *Anolis schiedii* group from Chiapas, Mexico. In: Johnson JD, Webb RG, Flores-Villela OA, editors. Mesoamerican herpetology: systematics, zoogeography, and conservation. Centennial Museum, special publication no. 1. El Paso: University of Texas at El Paso; 2000. p. 44–52.

28. Ponce-Reyes R, Reynoso-Rosales V-H, Watson JEM, VanDerWal J, Fuller RA, Pressey RL, et al. Vulnerability of cloud forest reserves in Mexico to climate change. Nat Clim Chang. 2012;2:448–52. doi: 10.1038/NCLIMATE1453
29. Pope I, Bowen D, Harbor J, Shao G, Zanotti L, Burniske G. Deforestation of montane cloud forest in the Central Highlands of Guatemala: Contributing factors and implications for sustainability in Q'eqchi' communities. Int J Sustain Dev World Ecol. 2015;22(3):201–12. doi: 10.1080/13504509.2014.998738
30. Rojas-Soto OR, Sosa V, Ornelas JF. Forecasting cloud forest in Eastern and Southern Mexico: conservation insights under future climate change scenarios. Biodivers Conserv. 2012;21(10):2671–90. doi: 10.1007/s10531-012-0327-x
31. Elsen PR, Monahan WB, Merenlender AM. Topography and human pressure in mountain ranges alter expected species responses to climate change. Nat Comm. 2020;11(1974). doi: 10.1038/s41467-020-15881-x
32. Ochoa-Ochoa LM, Mejía-Domínguez NR, Bezaury-Creel J. Priorización para la conservación de los bosques de niebla en México. Ecosistemas. 2017;26(2):27–37. doi: 10.7818/ECOS.2017.26-2.04
33. Myers N, Mittermeier RA, Mittermeier CG, da Fonseca GAB, Kent J. Biodiversity hotspots for conservation priorities. Nature. 2000;403(6772):853–8. doi: 10.1038/35002501
34. Toledo-Aceves T, Meave JA, Gonzalez-Espinosa M, Ramirez-Marcial N. Tropical montane cloud forests: current threats and opportunities for their conservation and sustainable management in Mexico. J Environ Manage. 2011;92(3):974-81. doi: 10.1016/j.jenvman.2010.11.007
35. Mittermeier RA, Gil PR, Hoffman M, Pilgrim J, Thomas B, Mittermeier CG, et al. Hotspots revisted: Earth's biologically richest and most endangered terrestrial ecoregions. Mexico City: Agrupación Sierra Madre, S.C.; 2004.
36. Gray JE. Catalogue of the slender-tongued saurians, with descriptions of many new genera and species, part 2. Annal Mag Nat Hist. 1838;Series 2(1):388–94.
37. Scarpetta SG, Ledesma DT. A strikingly ornamented fossil alligator lizard (Squamata: *Abronia*) from the Miocene of California. Zool J Linn Soc. 2023;197(3):752–67. doi: 10.1093/zoolinnean/zlac024
38. Gutiérrez-Rodríguez J, Zaldívar-Riverón A, Solano-Zavaleta I, Campbell JA, Meza-Lázaro RN, Flores-Villela O, et al. Phylogenomics of the Mesoamerican alligator-lizard genera *Abronia* and *Mesaspis* (Anguidae: Gerrhonotinae) reveals multiple independent clades of arboreal and terrestrial species. Mol Phylogenet Evol. 2021;154:106963. doi: 10.1016/j.ympev.2020.106963
39. Campbell JA, Frost DR. Anguid lizards of the genus *Abronia*: Revisionary notes, descriptions of four new species, a phylogenetic analysis, and key. Bull Am Mus Nat Hist. 1993;216:1–121.
40. Tihen JA. The genera of gerrhonotine lizards. Am Midl Nat. 1949;41(3):580–601. doi: 10.2307/2421775

41. Good DA. Phylogenetic relationships among gerrhonotine lizards: An analysis of external morphology. Univ Calif Publ Zool. 1988;121:1–139.
42. Chippindale PT, Ammerman LK, Campbell JA. Molecular approaches to phylogeny of *Abronia* (Anguidae: Gerrhonotinae), with emphasis on relationships in subgenus *Auriculabronia*. Copeia. 1998;1998(4):883–92. doi: 10.2307/1447335
43. Cope ED. Tenth contribution to the herpetology of Tropical America. Proc Am Philos Soc. 1877;17:85–98.
44. Clause AG, Luna-Reyes R, Nieto-Montes de Oca A. A new species of *Abronia* (Squamata: Anguidae) from a protected area in Chiapas, Mexico. Herpetologica. 2020;76(3):330–43. doi: 10.1655/Herpetologica-D-19-00047
45. García-Vázquez UO, Clause AG, Gutiérrez-Rodríguez J, Cazares-Hernández E, de la Torre Loranca MÁ. A new species of *Abronia* (Squamata: Anguidae) from the Sierra de Zongolica of Veracruz, Mexico. Ichthyol Herpetol. 2022;110(1):33–49. doi: 10.1643/h2021051
46. Sánchez-Herrera O, Solano-Zavaleta I, Rivera-Téllez E. Guía de identificación de los dragoncitos (lagartijas arborícolas, *Abronia* spp.) regulados por la CITES (PDF Navegable). CONABIO. México; 2017.
47. Güizado-Rodríguez MA, Porto-Ramírez SL. Los dragoncitos de México: lagartijas enigmáticas, desconocidas y amenazadas. Biodiversitas. 2018:2–6.
48. Hudson R, Sigler L, Guichard C, Flores O, Ellis S, editors. Conservatión, asesoramiento y manejo planificando para lagartijas *Abronia*. Informe. IUCN/SSC Conservation Breeding Specialist Group: Apple Valley, Minnesota. 2001.
49. Secretaría de Medio Ambiente y Recursos Naturales (SEMARNAT). Programa de Acción para la conservación de las especies *Abronia* (*Abronia* spp) en México. México: SEMARNAT/CONANP; 2018.
50. Hidalgo-García, JA, Luna-Reyes R, Clause AG, Carbajal-Márquez RA, Sigala-Rodríguez JJ, Muñoz-Alonso LA. Confirmation of the presence of the Striped Lizard Eater, *Mastigodryas dorsalis* (Bocourt, 1890) (Squamata, Colubridae), in Mexico. Check List 2023;19(1):115–125. doi: 10.15560/19.1.115
51. Bouzid NM, Rovito SM, Sanchez-Sólis JF. Discovery of the critically endangered Finca Chiblac salamander (*Bradytriton silus*) in Northern Chiapas, Mexico. Herpetol Rev. 2015;46(2):186–7.
52. Johnson JD, Mata-Silva V, García-Padilla E, Wilson LD. The herpetofauna of Chiapas, Mexico: composition, distribution, and conservation. Mesoam Herpetol. 2015;2(3):272–329.
53. Breedlove DE. Flora of Chiapas. Part 1. Introduction to the flora of Chiapas. San Francisco: The California Academy of Sciences; 1981.
54. Wake DB, Johnson JD. A new genus and species of plethodontid salamander from Chiapas, Mexico. Contr Sci Nat Hist Mus Los Angeles Co. 1989(411):1–10.
55. Luna-Reyes R, Pérez-López PE, García-Jiménez MA, Jiménez-Lang O, Gutiérrez-Morales OS, Cundapí-Pérez C, et al. Registros adicionales recientes, distribución potencial y

notas sobre el hábitat y ecología de la Salamandra Saltarina Negra *Ixalotriton niger* (Caudata: Plethodontidae). Lacandonia. 2015;9(1):65–78.
56. Mülleried GKF. Geología de Chiapas. Segunda edición. Tuxtla Gutiérrez: Colección Libros de Chiapas, Serie Básica, Gobierno del Estado de Chiapas; 1982.
57. Duellman WE. The hylid frogs of Middle America. Ithaca: Society for the Study of Amphibians and Reptiles; 2001.
58. Luna-Reyes R, Hernández-García E, Núñez-Orantes H. Anfibios y reptiles del Parque Educativo "Laguna Bélgica," Chiapas, México. Bol Soc Herpetol Mex. 2005;13(1):25–35.
59. Campbell JA. A new species of elongate *Abronia* (Squamata: Anguidae) from Chiapas, Mexico. Herpetologica. 1994;50(1):1–7.
60. Álvarez del Toro M. Los Reptiles de Chiapas. 2nd ed. Tuxtla Gutierrez: Instituto de Historia Natural del Estado. Departamento de Zoologia. Gobierno del Estado de Chiapas; 1973 "1972."
61. Grünwald CI, Pérez-Rivera N, Ahumada-Carillo IT, Franz-Chávez H, La Forest BT. New distributional records for the herpetofauna of Mexico. Herpetol Rev. 2016;47(1):85–90.
62. Álvarez del Toro M. Los Reptiles de Chiapas. 3rd ed. Tuxtla Gutiérrez: Instituto de Historia Natural del Estado. Departamento de Zoología; 1982.
63. Beaupre SJ, Jacobson ER, Lillywhite HB, Zamudio K. Guidelines for Use of Live Amphibians and Reptiles in Field and Laboratory Research. Second Edition, Revised by the Herpetological Animal Care and Use Committee (HACC) of the American Society of Ichthyologists and Herpetologists; 2004.
64. Eaton DAR, Overcast I. ipyrad: Interactive assembly and analysis of RADseq datasets. Bioinformatics. 2020;36(8):2592–94. doi: 10.1093/bioinformatics/btz966
65. Stamatakis A. RAxML version 8: a tool for phylogenetic analysis and post-analysis of large phylogenies. Bioinformatics. 2014;30(9):1312–13. doi: 10.1093/bioinformatics/btu033
66. Leaché AD, Banbury BL, Felsenstein J, Nieto-Montes de Oca A, Stamatakis A. Short tree, long tree, right tree, wrong tree: new acquisition bias corrections for inferring SNP phylogenies. Syst Biol. 2015;64(6):1032–47. doi: 10.1093/sysbio/syv053
67. Rambaut A. FigTree v. 1.4.4. 2018. https://github.com/rambaut/figtree/releases/tag/v1.4.4
68. Bradburd GS. conStruct: Models spatially continuous and discrete population genetic structure. Version 1.0.5. 2023. https://cran.r-project.org/package=conStruct
69. Bradburd GS, Coop GM, Ralph PL. Inferring continuous and discrete population genetic structure across space. Genetics. 2018;210(1):33–52. doi: 10.1534/genetics.118.301333
70. Leaché AD, Zhu T, Rannala B, Yang, Z. The spectre of too many species. Syst Biol. 2019;68(1):168–81. doi: 10.1093/sysbio/syy051
71. Yang Z. The BPP program for species tree estimation and species delimitation. Curr Zool. 2015;61(5):854–65. doi: 10.1093/czoolo/61.5.854
72. Flouri T, Jiao X, Rannala B, Yang Z. Species tree inference with BPP using genomic sequences and the multispecies coalescent. Mol Biol Evol. 2018;35(10):2585–93. doi: 10.1093/molbev/msy147

73. Yang Z, Rannala B. Bayesian species delimitation using multilocus sequence data. Proc Natl Acad Sci U.S.A. 2010;107:9264–69. doi: 10.1073/pnas.0913022107
74. Rambaut A, Drummond AJ, Xie W, Baele G, Suchard MA. Posterior summarisation in Bayesian phylogenetics using Tracer 1.7. Syst Biol. 2018;67(5):901–04. doi: 10.1093/sysbio/syy032
75. Bogert CM, Porter AP. A new species of *Abronia* (Sauria, Anguidae) from the Sierra Madre del Sur of Oaxaca, Mexico. Am Mus Novit. 1967;2279:1–21.
76. Campbell JA. A new species of *Abronia* (Sauria, Anguidae) from the Sierra Juárez, Oaxaca, México. Herpetologica. 1982;38(3):355–61.
77. Wiley EO, Mayden RL. The evolutionary species concept. In: Wheeler QD, Meier R, editors. Species concepts and phylogenetic theory: a debate. New York: Columbia University Press; 2000. p. 70–89.
78. Wiley EO, Mayden RL. A critique from the evolutionary species concept perspective. In: Wheeler QD, Meier R, editors. Species concepts and phylogenetic theory: a debate. New York: Columbia University Press; 2000. p. 146–58.
79. Wiley EO, Mayden RL. A defense of the evolutionary species concept. In: Wheeler QD, Meier R, editors. Species concepts and phylogenetic theory: a debate. New York: Columbia University Press; 2000. p. 198–208.
80. Wiens JJ, Servedio MR. Species delimitation in systematics: inferring diagnostic differences between species. Proc R Soc Lond B Biol Sci. 2000;267:631–6. doi: 10.1098/rspb.2000.1049
81. García-París M, Parra-Olea G, Brame Jr. II AH, Wake DB. Systematic revision of the *Bolitoglossa mexicana* species group (Amphibia: Plethodontidae) with description of a new species from México. Rev Esp Herpetol. 2002;16:43–71.
82. Castoe TA, Chippindale PT, Campbell JA, Ammerman LK, Parkinson CL. Molecular systematics of the Middle American Jumping Pitvipers (Genus *Atropoides*) and phylogeography of the *Atropoides nummifer* complex. Herpetologica. 2003;59(3):420–31. doi: 10.1655/01-105.2
83. Jadin RC, Townsend JH, Castoe TA, Campbell JA. Cryptic diversity in disjunct populations of Middle American Montane Pitvipers: a systematic reassessment of *Cerrophidion godmani*. Zool Scr. 2012;41(5):455–70. doi: 10.1111/j.1463-6409.2012.00547.x
84. Campbell JA, Solano-Zavaleta I, Flores-Villela O, Caviedes-Solis IW, Frost DR. A new species of *Abronia* (Squamata: Anguidae) from the Sierra Madre del Sur of Oaxaca, Mexico. J Herpetol. 2016;50(1):149–56. doi: 10.1670/14-162
85. Sánchez Álvarez M, Velasco Díaz N, García Álvarez M. Ote tzaʿʿmanhwajkuy. Vocabulario zoque. San Cristóbal de Las Casas, Chiapas: Fray Bartolomé de Las Casas, A.C; 2013. Spanish.
86. Cordry DB, Cordry DM. Trajes y tejidos de los Indios Zoques de Chiapas, México. Tuxtla Gutiérrez: Gobierno del Estado de Chiapas; 1988.
87. Najera-Lopez GG. Extracción de colorantes de líquenes y macromicetos de Chiapas [thesis]. Tuxtla Gutiérrez, Chiapas, México: Universidad de Ciencias y Artes de Chiapas; 2019.

88. Brindis-Segura O. Herpetofauna del Municipio de Coapilla, Chiapas, México [thesis]. Tuxtla Gutirérrez, Chiapas, México: Universidad de Ciencias y Artes de Chiapas; 2007.
89. Schmidt-Ballardo W, Solano-Zavaleta I, Clause AG. Nature notes. *Abronia deppii.* Reproduction. Mesoam Herpetol. 2015;2(2):192–4.
90. Solano-Zavaleta I, Cerón de la Luz NM, Clause AG. Solving a 50-year mystery: rediscovery of *Mesaspis antauges* (Squamata: Anguidae). Zootaxa. 2017;4303(4):559–72.
91. Aldape-López CT, Santos-Moreno A. *Abronia mixteca* (Mixtecan Arboreal Alligator Lizard). Mating behavior. Herpetol Rev. 2018;49(1):114.
92. Reyes G, Monzón J, Ariano-Sánchez D. Rediscovery after 48 years and geographic range extension of *Abronia anzuetoi* (Campbell & Frost, 1993) (Squamata: Anguidae) from Agua Volcano, Guatemala. Rev Lat Herpetol. 2022;5(4):108–111. doi: 10.22201/fc.25942158e.2022.4.554
93. Martínez-Coronel M, Ramírez-Bautista A, Vidal-López R. Geographic distribution. *Hyla bromeliacia* (Treefrog): México, Chiapas, Municipio Coapilla. Herpetol Rev. 1995;26(2):104–5.
94. Clause JK. Distribution notes. *Rhadinaea (Rhadinella) godmani* (Günther, 1865). Mexico, Chiapas, Municipio de Coapilla. Mesoam Herpetol. 2016;3(1):199.
95. Figueroa F, Sánchez-Cordero V. Effectiveness of Natural Protected Areas to prevent land use and land cover change in Mexico. Biodivers Conserv. 2008;17(13):3223–40. doi: 10.1007/s10531-008-9423-3
96. Sanfiorenzo-Barnhard C, García-Barrios L, Meléndez-Ackerman E, Trujillo-Vásquez R. Woody cover and local farmers' perceptions of active pasturelands in La Sepultura Biosphere Reserve buffer zone, Mexico. Mt Res Dev. 2009;29(4):320–7. doi: 10.1659/mrd.00013
97. García-Amado LR, Ruiz Pérez M, Barrasa García S. Motivation for conservation: assessing integrated conservation and development projects and payments for environmental services in La Sepultura Biosphere Reserve, Chiapas, Mexico. Ecol Econ. 2013;89:92–100. doi: 10.1016/j.ecolecon.2013.02.002
98. Cano-Díaz VC, Cortina-Villar S, Soto-Pinto L. La construcción de la acción colectiva en una comunidad del Área Natural Protegida: La Frailescana, Chiapas, México. Argumentos. 2015;28(77):79–95.
99. UNEP-WCMC, IUCN. Protected Planet: The World Database of Protected Areas (WDPA). Cambridge, UK: UNEP-WCMC and IUCN [cited 2022 Dec 12] Available from: www.protectedplanet.net
100. Periódico Oficial del Estado. Decreto por el que se declara Área Natural Protegida, con carácter de Zona Sujeta a Conservación Ecológica, el área conocida como "Tzama Cum Pümy." Periódico Oficial del Estado. Tomo II, No. 393. Decreto No. 432, 3 de noviembre de 2006. 2006.
101. Periódico Oficial del Estado. Decreto por el que se reforman y adicionan diversas disposiciones al decreto número 432, por el que se declara Área Natural Protegida, con carácter de Zona Sujeta a Conservación Ecológica, el área conocida como "Tzama Cum Pümy," ubicada en el municipio de Tapalapa, Chiapas. Periódico Oficial del Estado. Tomo III, No. 289. Decreto No. 180, 22 de marzo de 2011. 2011.

102. Clause AG, Solano-Zavaleta I, Soto-Huerta KA, de la A. Pérez y Soto R, Hernández-Jiménez CA. Morphological similarity in a zone of sympatry between two *Abronia* (Squamata: Anguidae), with comments on ecology and conservation. Herpetol Conserv Biol. 2018;13(1):183–93.
103. Moreno-Lara I, Cruz-Elizalde R, Suazo-Ortuño I, Ramírez-Bautista A. El tráfico de lagartijas emblematicas del género *Abronia* (Squamata: Anguidae). Rev Latinoam Herpetol. 2022;5(2):44–53. doi: 10.22201/fc.25942158e.2022.2.290
104. Solis N. California man pleads guilty to smuggling reptiles in his pants from Mexico into U.S. Los Angeles Times. 25 August 2022. Available from: https://www.latimes.com/california/story/2022-08-25/smuggling-wild-animals-united-states-mexico-border
105. Gluszek S, Ariano-Sánchez D, Cremona P, Goyenechea A, Luque Vergara DA, Mcloughlin L, et al. Emerging trends of the illegal wildlife trade in Mesoamerica. Oryx. 2020;55(5):708–16. doi: 10.1017/S0030605319001133
106. Tulloch AIT, Auerbach N, Avery-Gomm S, Bayraktov E, Butt N, Dickman CR, et al. A decision tree for assessing the risks and benefits of publishing biodiversity data. Nat Ecol Evol. 2018;2:1209–17. doi: 10.1038/s41559-018-0608-1
107. IUCN Standards and Petitions Committee. Guidelines for using the IUCN Red List Categories and Criteria. Version 14. Prepared by the Standards and Petitions Subcommittee; 2019 [cited 2022 Dec 12]. Available from: http://www.iucnredlist.org/documents/RedListGuidelines.pdf
108. Wilson LD, Mata-Silva V, Johnson JD. A conservation reassessment of the reptiles of Mexico based on the EVS measure. Amphib Reptile Conserv. 2013;7(1):1–47 (e61).
109. Secretaría de Medio Ambiente y Recursos Naturales (SEMARNAT). Norma Oficial Mexicana NOM-059-SEMARNAT-2010. Protección ambiental de especies nativas de México de flora y fauna silvestres. Categorías de riesgo y especificaciones para su inclusión, exclusión o cambio. Lista de especies en riesgo. 2010.
110. Sánchez-Salas J, Muro G, Estrada–Castillón E, Alba-Ávila JA. El MER: un instrumento para evaluar el riesgo de extinción de especies en México. Rev Chapingo Ser Zonas Áridas. 2013;12(1):30–5.
111. Pimm SL, Jenkins CN, Abell R, Brooks TM, Gittleman JL, Joppa LN, et al. The biodiversity of species and their rates of extinction, distribution, and protection. Science. 2014;344(6187):1246752. doi: 10.1126/science.1246752
112. Ripple WJ, Wolf C, Newsome TM, Hoffmann M, Wirsing AJ, McCauly DJ. Extinction risk is most acute for the world's largest and smallest vertebrates. Proc Nat Acad Sci USA. 2017;114(40):10679. doi: 10.1073/pnas.1702078114
113. Meiri S. Small, rare and trendy: traits and biogeography of lizards described in the 21st century. J Zool. 2016;299:251–61. doi: 10.1111/jzo.12356
114. Balderas-Valdivia CJ, González-Hernández A. Inventario de la herpetofauna de México 2021. Herpetol Mex. 2021;2:10–71.

115. Lara-Tufiño JD, Nieto-Montes de Oca A. A new species of Night Lizard of the genus *Lepidophyma* (Xantusiidae) from southern Mexico. Herpetologica. 2021;77(4):320–34. doi: 10.1655/Herpetologica-D-21-00019.1

116. McCranie JR, Matthews AJ, Hedges SB. A morphological and molecular revision of lizards of the genus *Marisora* Hedges & Conn (Squamata: Mabuyidae) from Central America and Mexico, with descriptions of four new species. Zootaxa. 2020;4763(3):301–53. doi: 10.11646/zootaxa.4763.3.1

117. Butler BO, Smith LL, Flores-Villela O. Phylogeography and taxonomy of *Coleonyx elegans* Gray 1845 (Squamata: Eublepharidae) in Mesoamerica: the Isthmus of Tehuantepec as an environmental barrier. Mol Phylogenet Evol. 2023;178:107632. doi: 10.1016/j.ympev.2022.107632

118. Solano-Zavaleta I, Nieto-Montes de Oca A. Species limits in the Morelet's alligator lizard (Anguidae: Gerrhonotinae). Mol Phylogenet Evol. 2018;120:16–27. doi: 10.1016/j.ympev.2017.11.011

119. Valdenegro-Brito AE, Pavón-Vázquez CJ, Luna-Reyes R, García-Vázquez UO. Distribución geográfica de *Scincella incerta* (Squamata: Scincidae) en el estado de Chiapas, México. Acta Zool Mex (n s). 2018;34:e3412140. DOI: 10.21829/azm.2018.3412140

120. Hidalgo H. Two new species of *Abronia* (Sauria: Anguidae) from the cloud forests of El Salvador. Occ Pap Mus Nat Hist Univ Kansas. 1983(105):1–11.

121. García-Padilla E, Escalante-Pliego P. Depredación de lagartijas *Abronia* por el quetzal mesoamericano (*Pharomachrus mocinno*). Huitzil Rev Mex Ornitol. 2022;23(2):e-638. doi: 10.28947/ hrmo.2022.23.2.668

122. Martínez-Coronel M, Vidal-López R. Nota de distribución de dos murciélagos molósidos en Chiapas, México. Vert Mex. 1997;4:17–9.

123. Vidal-López R. Los mamíferos de Coapilla, Chiapas, México [thesis]. Tuxtla Gutiérrez, Chiapas, México: Universidad de Ciencias y Artes del Estado de Chiapas; 1998.

124. Rubiano A. MP. The tale of one tiny songbird is amplifying an ancient Mayan language [cited 2022 Dec 12] Available from: https://www.audubon.org/news/the-tale-one-tiny-songbird-amplifying-ancient-mayan-language

125. Cayuela L, Golicher DJ, Rey-Benayas JM. The extent, distribution, and fragmentation of vanishing Montane Cloud Forest in the highlands of Chiapas, Mexico. Biotropica 2006;38(4):544–554. doi: 10.1111/j.1744-7429.2006.00160.x

126. Johnson JD, Mata-Silva V, Ramírez-Bautista A. Geographic distribution and conservation of the herpetofauna of Southeastern Mexico. In: Wilson LD, Townsend JH, Johnson JD, editors. Conservation of Mesoamerican amphibians and reptiles. Eagle Mountain: Eagle Mountain Publishing, LC; 2010. p. 323–69.

127. Montiel Canales G, Mayer Goyenechea IG. Amphibian areas of endemism: a conservation priority in the threatened Mexican cloud forest. Vertebr Zool. 2022;72:235–244. doi: 10.3897/vz.72.e73534

128. Ochoa-Ochoa L, Urbina-Cardona JN, Vázquez L-B, Flores-Villela O, Bezaury-Creel J. The effects of governmental protected areas and social initiatives for land protection on the conservation of Mexican amphibians. PLoS ONE. 2009;4(9):1–9. doi: 10.1371/journal.pone.0006878
129. Méndez-López, ME, García-Frapolli E, Pritchard DJ, Sánchez González MC, Ruiz-Mallén I, Porter-Bolland L, Reyes-Garcia V. Local participation in biodiversity conservation initiatives: a comparative analysis of different models in South East Mexico. J Environ Manage. 2014;145:321–329. doi: 10.1016/j.jenvman.2014.06.028
130. Jareguiberry P, Titeux N, Wiemers M, Bowler DE, Coscieme L, Golden AS, Guerra CA, Jacob U, Takahashi Y, Settele J, Díaz S, Molnár Z, and A. Purvis A. The direct drivers of recent global anthropogenic biodiversity loss. Sci Adv. 2022;8(45):eabm9982. doi: 10.1126/sciadv.abm9982
