# Supplementary material for: Bridging the gap: A new species of arboreal Abronia (Squamata: Anguidae) from the Northern Highlands of Chiapas, Mexico
Source: PLoS One. 2024 Jan 3;19(1):e0295230. doi: 10.1371/journal.pone.0295230 (PMC10763973; doi:10.1371/journal.pone.0295230)
Supplement: Attachment — Submitted filename: Comments.docx [file pone.0295230.s004.docx]

Comments

Dear Editor,

I have read the MS entitled “Bridging the gap: A new species of arboreal *Abronia* (Squamata: Anguidae) from the Northern Highlands of Chiapas, Mexico” by Adam G. Clause, Roberto Luna-Reyes,¶ Oscar M. Mendoza-Velázquez¶, Israel Solano-

Zavaleta.

This a good paper, description of a new species of *Abronia*; it is well written, good material, and the species is described based in morphological characteristics (diagnostic characteristics), from my point of view, the use of morphological methods for describing new species, it is good as long as the sample size indicates the diagnostic characters that indicate that they represent a new species and this is shown here; otherwise the authors would have to use molecular methods (molecular biology). Therefore, my comments on this work are very soft, indicating some suggestions that the authors can attend to if they wish.

Title- why Bridging the gap? Could you explain it a little bit in introduction?

Introduction. Could you explain a little more about the Mesoamerican region in terms of it being a center of diversification and its importance from a biogeographical point of view, its endemicity. The importance of cloud forest and pine-oak forest environments, which are the environments in which *Abronia* species inhabit. It is important to give it a sense of conservation and protection to maintain biodiversity (see comments in text).

Methods- Holotype- The morphological part that comes from the holotype is very heavy, perhaps mentioning the diagnostic characters that indicate being a new species will be enough and comparing these with the characters of another or other species closer to the genus *Abronia* would be enough, and in a section (appendix) you can put the other characters that are important to recognize the new species, but you could solve in this way putting a large number of characters that the reader is lost (it is similar en results section).

Results- see comments on text.

Discussion- See comments on text. It is important to point out the care of the environment in these areas where the diversification of the species occurs, focusing on the conservation of biodiversity, specifically in the genus *Abronia*, whose species are arboreal; in these environments, the anthropic effect is ending the vegetation cover, eliminating the refuge(s) of the species.
